# Exploring the Conformational Transitions of Biomolecular Systems Using a Simple Two-State Anisotropic Network Model

**DOI:** 10.1371/journal.pcbi.1003521

**Published:** 2014-04-03

**Authors:** Avisek Das, Mert Gur, Mary Hongying Cheng, Sunhwan Jo, Ivet Bahar, Benoît Roux

**Affiliations:** 1 Department of Biochemistry and Molecular Biology, Gordon Center for Integrative Science, University of Chicago, Chicago, Illinois, United States of America; 2 Department of Computational & Systems Biology, School of Medicine, University of Pittsburgh, Pittsburgh, Pennsylvania, United States of America; Max Planck Institute for Biophysical Chemistry, Germany

## Abstract

Biomolecular conformational transitions are essential to biological functions. Most experimental methods report on the long-lived functional states of biomolecules, but information about the transition pathways between these stable states is generally scarce. Such transitions involve short-lived conformational states that are difficult to detect experimentally. For this reason, computational methods are needed to produce plausible hypothetical transition pathways that can then be probed experimentally. Here we propose a simple and computationally efficient method, called *ANMPathway*, for constructing a physically reasonable pathway between two endpoints of a conformational transition. We adopt a coarse-grained representation of the protein and construct a two-state potential by combining two elastic network models (ENMs) representative of the experimental structures resolved for the endpoints. The two-state potential has a cusp hypersurface in the configuration space where the energies from both the ENMs are equal. We first search for the minimum energy structure on the cusp hypersurface and then treat it as the transition state. The continuous pathway is subsequently constructed by following the steepest descent energy minimization trajectories starting from the transition state on each side of the cusp hypersurface. Application to several systems of broad biological interest such as adenylate kinase, ATP-driven calcium pump SERCA, leucine transporter and glutamate transporter shows that *ANMPathway* yields results in good agreement with those from other similar methods and with data obtained from all-atom molecular dynamics simulations, in support of the utility of this simple and efficient approach. Notably the method provides experimentally testable predictions, including the formation of non-native contacts during the transition which we were able to detect in two of the systems we studied. An open-access web server has been created to deliver *ANMPathway* results.

## Author Summary

Many biomolecules are like tiny molecular machines that need to change their shapes and visit many states to perform their biological functions. For a complete molecular understanding of a biological process, one needs to have information on the relevant stable states of the system in question, as well as the pathways by which the system travels from one state to another. We report here an efficient computational method that uses the knowledge of experimental structures of a pair of stable states in order to construct an energetically favoravle pathway between them. We adopt a simple representation of the molecular system by replacing the atoms with beads connected by springs and constructing an energy function with two minima around the end-states. We searched for the structure with highest energy that the system is most likely to visit during the transition and created two paths starting from this structure and proceeding toward the end-states. The combined result of these two paths is the minimum energy pathway between the two stable states. We apply this method to study important structural changes in one enzyme and three large proteins that transport small molecules and ions across the cell membrane.

## Introduction

Complex macromolecular systems such as enzymes, channels, transporters and pumps need to change their shapes and visit many conformational states in order to perform their functions. Experimental data from functional, biochemical, spectroscopic and structural techniques often inform us on the long-lived stable functional states of macromolecular systems. Accordingly, the average structures of thousands of important biomolecules have been determined using X-ray crystallography or NMR. For many well-studied systems, hundreds of structures have been resolved in the presence of different ligands, or under different conditions or functional states. In contrast, for most systems, little or no experimental data are often available on the intermediate structures along the conformational transition pathway associated with a function. To understand the molecular mechanism of a specific biological process, one needs to go beyond the static information and determine how macromolecules change their conformations as a function of time. In practice, however, obtaining direct structural data about a transition pathway is exceedingly difficult, because intermediate conformations are transient and usually short-lived compared to the timescale of the whole process.

Computational methods can help generate physically plausible pathways for conformational transitions, which can then serve as “hypotheses” to be tested and refined experimentally [1]–[3]. The relevance of any *in silico* pathway lies in its ability to predict the occurrence of intermediates, which can sometimes be detected using X-ray crystallography [1] or indirectly inferred by perturbing the system via site-directed mutagenesis [3]. Computation and experimental validation thus offers a powerful combination to study the mechanisms of complex biomolecular events.

All-atom molecular dynamics (MD) simulation, arguably, provides the most realistic representation of biomolecular dynamics [4], [5]. If one could simulate the system of interest for sufficiently long time-scales then the trajectory could provide the information required to understand a conformational transition (albeit based on a virtual model). However, brute-force MD is often impractical since most large-scale conformational changes take place over timescales ranging from milliseconds to seconds, which are far beyond the reach of the most powerful supercomputers. Special-purpose hardware and software like Anton [6] are pushing the limits of current molecular simulations; however, they still fall short of accessing the relevant time-scales for the cooperative structural changes of large biological systems. Statistical mechanical methods have also been developed specifically to simulate rare dynamical events [7]–[10], though their application to study large-scale conformational transitions in biological macromolecules remains challenging.

One alternative strategy has been to formulate the problem of the conformational reaction pathway as a “chain-of-state”, i.e., a sequence of configurations representing the progress of the system between two known end-states in the multi-dimensional conformation space [11]–[15]. For example, the so-called “string method” based on all-atom MD simulations has been employed successfully to study functionally important conformational transitions in a variety of biological systems, including Src kinases [16], insulin receptor kinase [17], [18], adenylate kinase [19], amyloidogenic isomerization of 2-microglobulin [20], cholesterol flip in membranes [21], myosin VI [22], DNA polymerase [23], and voltage-gated K^+^ channels [3]. Other notable methods that seek to shed some light on the important intermediate structures between experimentally known intermediates include the weighted ensemble method [24]–[27] and dynamic importance sampling [28]–[30]. Several enhanced sampling methods such as conformational flooding [31], metadynamics [32] and accelerated molecular dynamics [33] have also been used for similar purpose even though these methods are not designed for searching for transition pathways between two known endpoints. A very different approach based on shapes of biomolecules rather than detailed energetics of the system has been used to search for transition pathways in the tCONCOORD method of Seeliger *et al.* [34]. However, despite these promising advances, the investigation of large-scale transitions of multimeric systems at atomic details remains prohibitively expensive. It is, therefore, desirable to dispose of simpler models and computationally efficient methods to generate a putative pathway with qualitatively reasonable features that can be tested by experiments.

A straightforward way to reduce the computational cost is to simplify the atomistic system by constructing a coarse-grained (CG) model and adopting a simple potential function that uses knowledge of resolved structures. We adopt here a broadly used/tested structure-based CG model, the elastic network model (ENM) [35]–[37], a powerful example of which is the anisotropic network model (ANM) [38].

In the ANM, the protein is represented by a set of CG sites (nodes) placed at the positions of 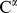 atoms of all the residues and the energy function is a pairwise additive harmonic potential where each site interacts with all the sites within a cut-off distance. ENMs are often used in conjunction with normal mode analysis (NMA) where one diagonalizes the Hessian matrix of the potential constructed around an experimental structure and studies the deformation of the system along the low frequency normal modes. The simplified potential function in the ANM presents the advantage of yielding an analytical expression for the Hessian, directly expressed in terms of the known structure coordinates [38], which is readily decomposed to obtain the ANM (normal) modes. It has been found for a wide variety of large biomolecular systems that collective motions relevant to function occur along the low energy normal modes of motions predicted by ENMs [39]–[45], suggesting that native contact topology accounted for by the network model is a major determinant of accessible modes of function.

Even though ENMs coupled with NMA have been successful in providing insights into important conformational transitions, they explore, by definition, the neighborhood of a given energy minimum and as such they are not adequate for constructing a transition pathway between two endpoints (minima on conformational energy landscape). However, ENM-based approaches have been very influential in the development of a series of methods that aim at providing plausible intermediate structures along a transition. One of the early studies along these lines is that of Jernigan, Chirikjian and coworkers [46], [47] who have used an interpolation technique with distance constraints to avoid steric clashes. They also showed that normal mode calculations could be accelerated by dividing the system into rigid clusters connected by elastic springs [48], and employed cluster-NMA for constructing pathways by successively creating new structures from an end-state [49], [50]. Miyashita *et al.* [51] started from one stable state, performed successive normal mode calculations and for each new set of normal modes used a small subset based on the overlap with the other end structure to create an intermediate structure. In the plastic network model (PNM), Maragakis and Karplus [52] constructed a two-state elastic network potential by mixing two ENMs, one for each end-state, and then the pathway was constructed in two steps: identification of a saddle point and two steepest descent minimizations. Yang *et al.* [53] used the same two-state potential to start from both end structures and used well-defined criteria for recruiting small subsets of normal modes to create a series of intermediate conformers via an *a*daptive ANM (*a*ANM) methodology until the two intermediates merged within a predefined root-mean-square-deviation (RMSD). Hummer and co-workers [54]–[56] also constructed a two-state potential by mixing two ENM surfaces using an exponential mixing rule and constructed the pathway on this surface using saddle point search. Yang and Roux [57] have used a two-state G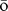 model [58], [59] and extensive CG simulations in conjunction with clustering methods to investigate pathways in conformational transition of Src-kinase. Chu and Voth [60] used a more complicated two-state potential by representing each pairwise interaction as a double well and used a saddle point search algorithm to construct the pathway. Their double-well network model has more frustration than a two-state elastic network model and captures complexity of a transition that are not present in models with smoother potential energy functions. Franklin *et al.* [61] used two ENM surfaces in an entirely different way to construct a pathway method. In their MinActionPath method, they developed an algorithm based on the minimization of the Onsager-Machlup action to construct the path with minimum resistance between two stable states. Even though the problem of construction of pathway between two stable states described by simple CG models has attracted a lot of attention, there is still enough room and need for development of new methods that can address the scalability problem in particular, and help efficiently calculate pathways for large systems.

We propose a simple and efficient method, called *ANMPathway*, and apply the method to understand conformational transitions of several important globular and membrane proteins. We adopt a simple ENM representation for each of the end-states, which accounts for the topology of inter-residue contacts in the structure. We construct a very simple two-state potential by mixing these two ENMs. Our potential has a cusp hypersurface where the energies from both the ENMs are same. We search for a minimum energy structure on the cusp hypersurface and treat it as the transition state. We then start from the transition state and perform two separate steepest descent minimizations to connect the end-states. Conformers collected from two steepest descent paths along with the transition state provide a pathway. Even though the existence of a cusp hypersurface in our potential is somewhat unphysical, we demonstrate, by way of applications to several systems (adenylate kinase (AK), ATP driven calcium pump SERCA, leucine transporter (LeuT) and glutamate transporter (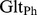)), that *ANMPathway* gives physically meaningful pathways and helps generate experimentally testable hypotheses.

## Methods

The goal of the *ANMPathway* method is to construct a transition pathway between two end-states of a conformational transition. As in the string method, the pathway is represented by a chain of equidistant states (conformers/images) [11]–[14]. The macromolecular structure is described by a CG model where interaction sites are placed at the positions of 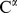 atoms, which serve as the set of collective variables for the string. Conceptually, the string is the minimum free energy pathway on the potential of mean force (PMF) of the system with respect to those collective variables [14]. Assuming a Euclidian metric in the cartesian space of the 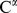 atoms, the equidistant conditions implies that neighboring images along the string are separated by a fixed RMSD. However, in practice, the ENM energy function employed in the CG model is a knowledge-based construct that is only applicable near the experimental structure used to construct the model. For describing a conformational transition between two stable states, we need an approximation to the PMF that is applicable for large distortions from the experimental structures.

In the presence of structural data on the end-states, it is reasonable to construct an effective energy function with two minima centered around the endpoints of the transition. One obvious route to such two-state potentials involves creating two separate energy surfaces that are defined around each of the end-states and then combine these surfaces by an empirical rule. We have adopted this strategy and used two ANMs [38] and a very simple mixing rule to construct an energy function with two minima.

For a protein with *N* residues, the configuration of the system is denoted by a 3*N* dimensional vector 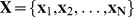 where, 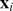 is a three-dimensional vector giving the position of the *i*th site (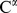 atom of the *i*th residue). ANM is an elastic network model defined around an experimental structure (e.g. crystal or NMR structure) 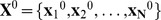 with the following energy function
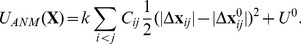
(1)Here 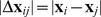 is the distance between nodes *i* and *j*, *k* is the uniform force constant, *C_ij_* is an element of the contact matrix defined by
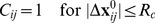
(2)

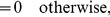
(3)

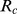
 is the cut-off distance and 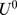 is the energy of the system at the reference state. The advantage of including the 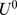 term is that it allows us to create energy difference between the end-states when more than one ENM are included in the model. In order to construct the potential function we first define two ANM energy functions, 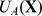 and 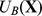, centered around the end structures 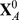 and 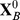 and combine them by the following mixing rule,
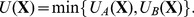
(4)The energy difference between the end-states of this two-state ENM is 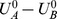.

The two-state potential based on Eq. (4) has a cusp hypersurface in the 3*N*-dimensional configuration space. Even though potential energy functions developed for real systems are differentiable everywhere, we will show that the simple two-state potential is a reasonable first approximation and is capable of capturing important qualitative features of the conformational transition in question. Both the ANM energy functions are 3*N*-dimensional harmonic surfaces and the hypersurface where they intersect (i.e. where the energies from both ANM surfaces are same) is another harmonic surface of dimension 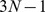. We define the transition state as the minimum energy structure on the cusp hypersurface. Given a sequence of conformers that linearly interpolates the Cartesian distance between two conformers that reside on the opposite sides of the cusp hypersurface, it is possible to identify a conformer that has equal energies, within a tolerance, from both the ANM surfaces. This conformer, by construction, resides on the cusp hypersurface. This simple observation allows us to devise an algorithm to search for the energy minimum on the cusp hypersurface i.e. the transition state. Once we have identified the transition state, we can start from there and slide down the harmonic surfaces until we reach the endpoints, by performing two separate steepest descent minimizations. In the end, we collect all the conformers in proper order to construct the transition pathway. The pathway obtained by *ANMPathway* can be regarded as the minimum energy path between the end structures since it is the combination of two steepest descent paths on two surfaces joined at the transition state which is the minimum energy conformer on the cusp hypersurface. A detailed description of the algorithm is given below.

1. Two end structures are represented by the positions of their 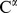 atoms. These structures are aligned and 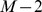 new intermediate conformers/images are generated by linearly interpolating between the end structures. The value of *M* is chosen by the user and is dependent on the value of the tolerance parameter 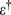, which is the smallest energy difference between two conformers that are considered to be different.
2. For each image the energy is determined using the two-state potential defined in Eq. (4). We identify the conformer 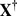 for which energies from both the surfaces (i.e. 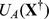 and 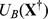) are equal within the tolerance parameter 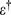.
3. Starting from 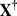 the transition state is searched by the following iterative procedure:
  - With appropriate choices of step-sizes 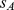 and 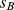 and knowledge of transition state for the present iteration 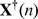, one step of steepest descent minimization is carried out on each surface using the force of the respective surface and two new sets of coordinates, 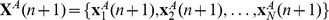 and 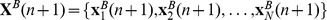 are generated, where
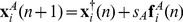
(5)

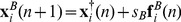
(6)and
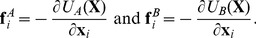
(7)
  - A linear interpolation is performed between 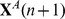 and 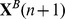 to find out the conformer that resides on the cusp hypersurface. This is the new approximation for transition state i.e. 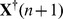

  - We iterate steps (3a) and (3b) until the energy difference between two transition state conformers, obtained in two successive iterations, is less than a tolerance 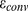.
4. Two separate steepest descent minimizations are performed, one on each surface, starting from the final transition state conformation 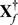 and conformers separated by a user-defined RMSD are collected.
5. Conformers are indexed in the following sequence to construct a pathway: end structure *A*, conformers collected on surface *A* with increasing RMSD from the end structure *A*, transition state conformer 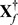, conformers collected on surface *B* with decreasing RMSD from the end structure *B*, end structure *B*.

Several parameters listed above need to be specified before performing the calculation. The two-state potential function is characterized by the force constants and cut-off distances of ANMs. The ANM force constant does not affect the qualitative results (or the shape of conformational change driven by the normal modes), but uniformly scales the absolute size of motions. Our choice of force constants is inconsequential, since the absolute size of the motion is adjusted by the step sizes 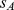 and 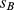. The cut-off distance is usually selected in the range 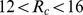 Å and the overall qualitative features of the pathways were found to be quite insensitive to the choice of 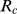 within this range for all the systems we studied. If desired the force constants can be estimated by fitting the crystallographic B-factors although the B-factors themselves may be biased by the crystallization conditions and crystal contacts. The energy offsets can be tuned if there are experimental information on the relative energies of the end-states. The value of 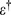 was chosen to be in the range between 10^−4^ and 10^−5^ which could be achieved by setting *M* = 100 (step 1 of the algorithm). The most important parameters for an efficient implementation of the algorithm turned out to be the step-sizes involved in the transition state search on the cusp hypersurface (*s_A_* and *s_B_* in step 3a). If step-sizes are too large then the resultant movement of the transition state structure on the cusp hypersurface is large and the minimization algorithm does not work. On the other hand, if the chosen values are too small then the convergence becomes slow. For optimal values of step-sizes, short trial runs were performed for several choices, starting from large values and systematically decreasing them at each trial run until the energy of the transition state conformer decreased monotonically for the entire duration of the trial run. The starting values of *s_A_* and *s_B_* were chosen between 0.8 and 0.4 with ANM force constants set at 0.1 kcal/(mol Å^2^). These values need to be adjusted if the force constants are changed by maintaining the inverse proportionality between the step-size and the force constant. Our experience shows that a few very short trial runs are sufficient for finding the optimal values of *s_A_* and *s_B_* and the overall procedure is extremely efficient. The convergence criterion 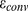 was selected between 10^−4^ and 10^−5^. The number of iterations needed for convergence ranged from 200 to 1000 for the systems studied in this paper.

The pathway is constituted of equally spaced structures obtained by the above mentioned algorithm between the two end structures. We have calculated several quantities to analyse the pathway and understand the conformational transition in terms of collective coordinates. The change in energy of the system along the pathway illustrates the shape of the harmonic surfaces used to describe the system. For example, if the structural change involves movements along the low frequency modes, the energy changes are smaller for a given deformation, compared to those involved in movements along high frequency (more local) movements. The cusp region along the pathway, which is easily identified as the place where the system hops from one surface to another, does not necessarily fall in the middle of the two end structures if one endpoint is more compact than the other. In order to understand the importance of the normal modes (ANM modes) of one end structure in describing the conformational transition, we calculated the cumulative correlation cosine, defined below, of few selected structures along the pathway
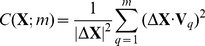
(8)where 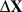 is the displacement of a selected conformer/image from one of the reference end structures, 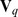 is the *q*th normal mode of that structure and *m* is the total number of modes (starting from the lowest frequency modes) used for evaluating the cumulative correlation cosine.

It is difficult to validate the results of our method with direct experimental observations. Many of the intermediate structures in a pathway are short-lived and may not be amenable to experimental detection. However, it is reasonable to expect that some predictions can be indirectly verified. In order to make closer connections to experiments, we have looked at the possible formation of close non-native contacts along the pathway. The hope is that some of these predictions can be tested in cross-linking experiments. We looked for pairs of residues that are far apart (>10 Å) in both the native states but come close (<5–7 Å) somewhere along the pathway. We were able to find such pairs in two of the four systems we studied.

## Results

### Adenylate Kinase

**Figure 1:**
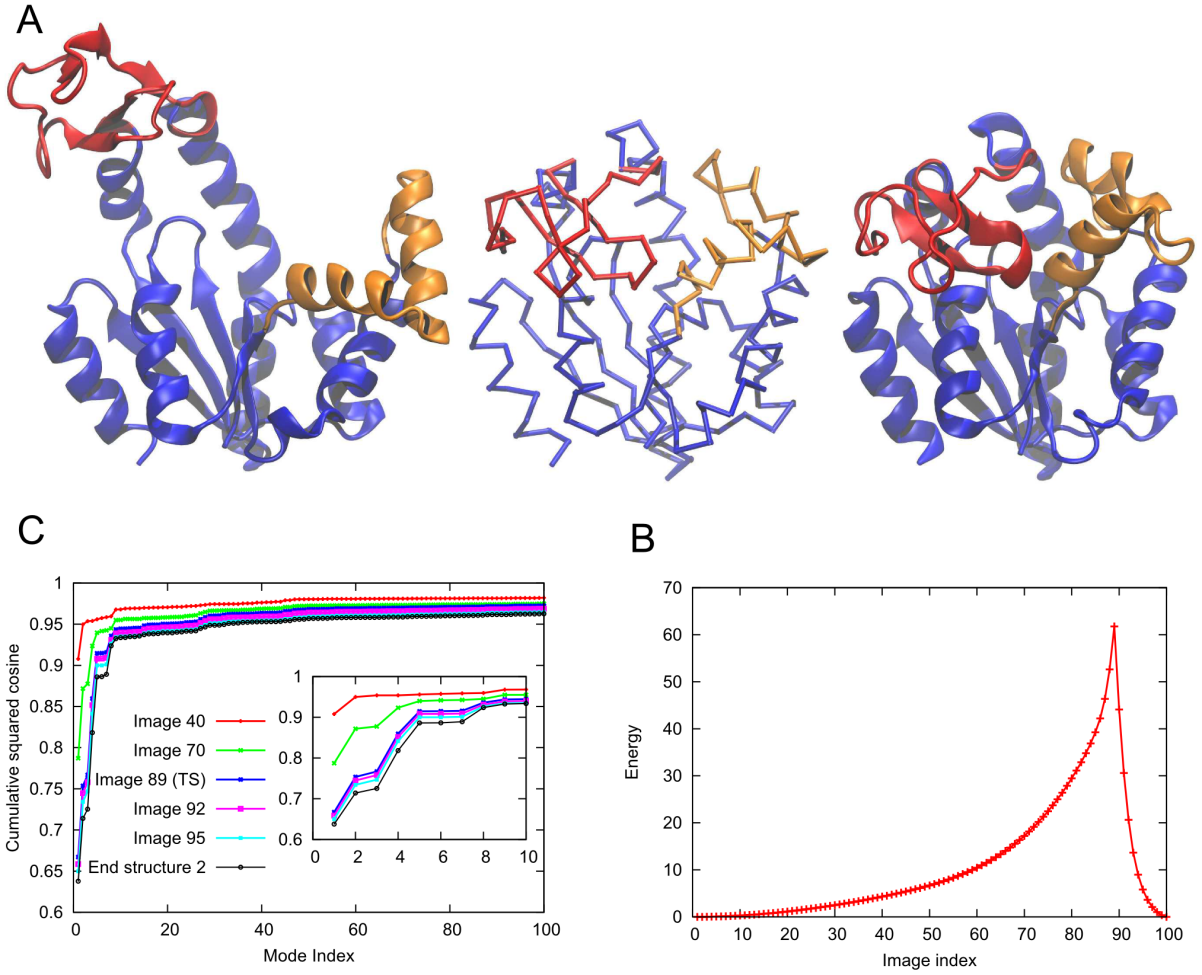
Conformational transition between the open and the closed states of adenylate kinase. **A.** Structures of the open (*left*, PDB ID: 4AKE) and the closed (*right*, PDB ID: 1AKE) states. The LID and the NMP domains are shown in *red* and *orange* respectively. The CORE domain and the rest of the protein are shown in *blue*. The central structure is the 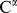 trace of the transition state produced by *ANMPathway*. **B.** The energy of the system along the transition. Total number of images in the pathway is 100, RMSD between two consecutive images is ∼0.1 Å. The transition state corresponds to image 89. **C.** Cumulative squared cosines between ANM modes and the change in structure between the initial state and a few selected conformers (images) along the transition pathway. The modes were calculated for the starting structure (open state). Here and in the counterparts generated for other test cases, the lowest frequency (slowest modes) end of the graph is enlarged in the inset. The force constants and cut-offs for both the end-states were set to 0.1 kcal/(mol Angstrom) and 15 Å in all applications in the present study, and no energy offsets were used for either of the end structures.

Adenylate kinase (AK) is an enzyme that catalyzes the transfer of a single phosphoryl group from ATP to AMP via the reversible reaction 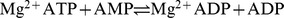. The structure of AK consists of three domains: the AMP-binding domain (NMP), the ATP-binding domain (LID) and the CORE domain (Fig. (1A)). The phosphoryl transfer reaction involves a large-scale conformational transition in AK. In the open (O) state, the NMP and LID are farther apart; and in the closed state, they are tightly packed (right and left structures in Fig. (1A)). We have applied *ANMPathway* on the open (O) to closed (C) transition in AK. The end-states were obtained from the crystal structures (PDB IDs: 4AKE [62] and 1AKE [63] for the O and C states, respectively). The pathway has 100 images with an RMSD of 0.1 Å between two consecutive images and the transition state corresponds to image 89 (Fig. (1B)) which is situated almost at the end of the open to close transition. The transition between the functional substates of AK comprises large scale hinge-like motions of NMP and LID with respect to a rigid CORE. At the initial stage only the LID moves like a rigid body and the rest of the protein is almost unchanged. This motion corresponds to the slow rise in energy (Fig. (1B)). Then the NMP starts to move and the energy rises as local structural rearrangements take place. Finally the CORE domain undergoes some changes and the transition is complete (movie S1 in Supplementary Material (SM)). The overall result is a two step transition mechanism: LID closing followed by NMP closing (or in the reverse direction: NMP opening followed by LID opening).

Because of the functional importance of domain opening/closing, it is natural to expect that the transition can be described by a small number of normal modes, as shown in Fig. (1C). For image 40, only two modes are sufficient to represent 90% of the displacement from the starting (O) structure. As we move away from the reference state, more modes are needed but the number of modes increases slowly. For attaining the other end structure (C, black curve) with a correlation cosine of more than 0.90 no more than 10–15 modes are needed, which is only ∼2% of the total number of available modes. Therefore the normal mode picture is extremely useful for studying this transition.

**Figure 2:**
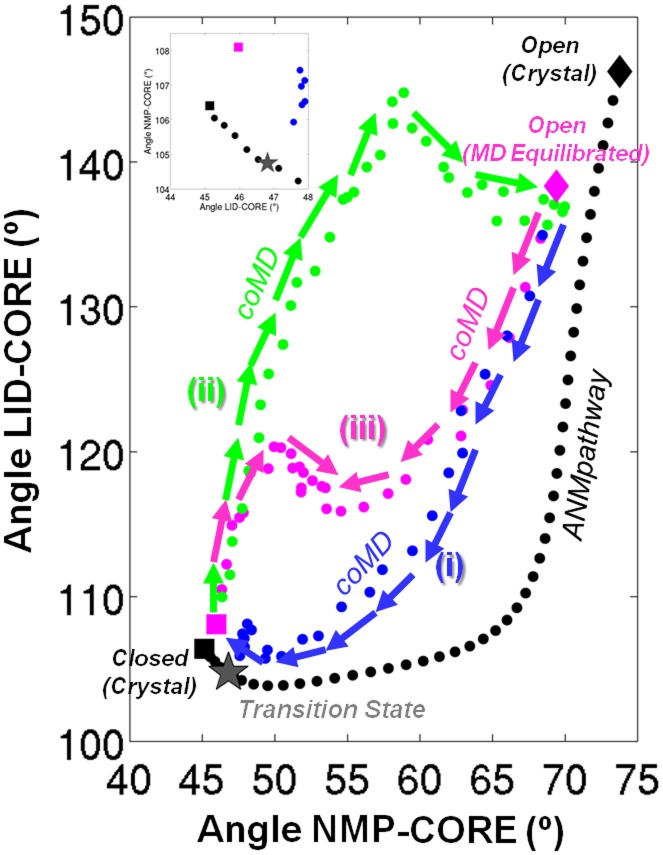
Transition pathways displayed as a function of LID-CORE and NMP-CORE angles. Closed (*bottom*, *square*) and Open (*top*, *diamond*) substates of AK for the crystal structures (*black*) and their MD equilibrated (*magenta*) conformers. *ANMPathway* and three *coMD* [64] pathways are depicted as *black*, *blue*, *magenta* and *green* dots, respectively, with the arrows indicating the direction of reconfiguration in each case. The relative positions of the domains are defined similar to Beckstein *et al.* [72] and Gur *et al.* [64]. The NMP-CORE angle is the angle between the centers of mass of three segments I90-G100, L115-V125 and L35-A55, based on 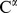 atoms and the LID-CORE angle is the angle between centers of mass of 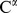 atoms of L115-V125, I179-E185 and V125-L153.

In recent years, several computational studies have examined the 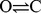 allosteric transition in AK. These studies revealed a multiplicity of pathways, as well as their dependence on the initial conformers. Among them, two types of transitions appear to be consistently observed in independent studies: (i) LID closing followed by NMP closing along the 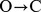 transition [52], [64]–[71] and (ii) LID opening followed by NMP opening along the 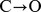 transition [64], [70]–[72]. Fig. (2) illustrates these pathways in the conformational space defined by LID-CORE and NMP-CORE angles (see Fig. (2) caption for the definition of the angles), with the intermediates obtained by a recently introduced hybrid methodology, *coMD* [64], and by *ANMPathway*, as labeled. As can be seen, mechanisms (i) and (ii) are predicted by *coMD* provided that the starting points are the O and C states, respectively. The pathway (iii), on the other hand, is obtained by conducting two parallel runs, starting from both ends, and generating intermediates until the two paths merge. The initial steps conform to paths (i) and (ii) in this case. Similar transition mechanisms were recently reported by Kidera and coworkers [19] in further support to the LID-movement-first behavior in both directions. This behavior is also in agreement with the free energy surface obtained by Woolf and coworkers [72] as a function of LID-CORE and NMP-CORE angles, although other mechanisms, such as NMP-first closure for unligated AK [67] or monotonous LID/NMP closing [72] have also been reported. *ANMPathway* yielded a transition state very close to the closed state, i.e. by definition, most of the change in structure (89 steps out of 100) proceeded in the energy well near the open state, hence its consistency with modes accessible to end-state O, or pathway (i).

### Leucine Transporter (LeuT)

**Figure 3:**
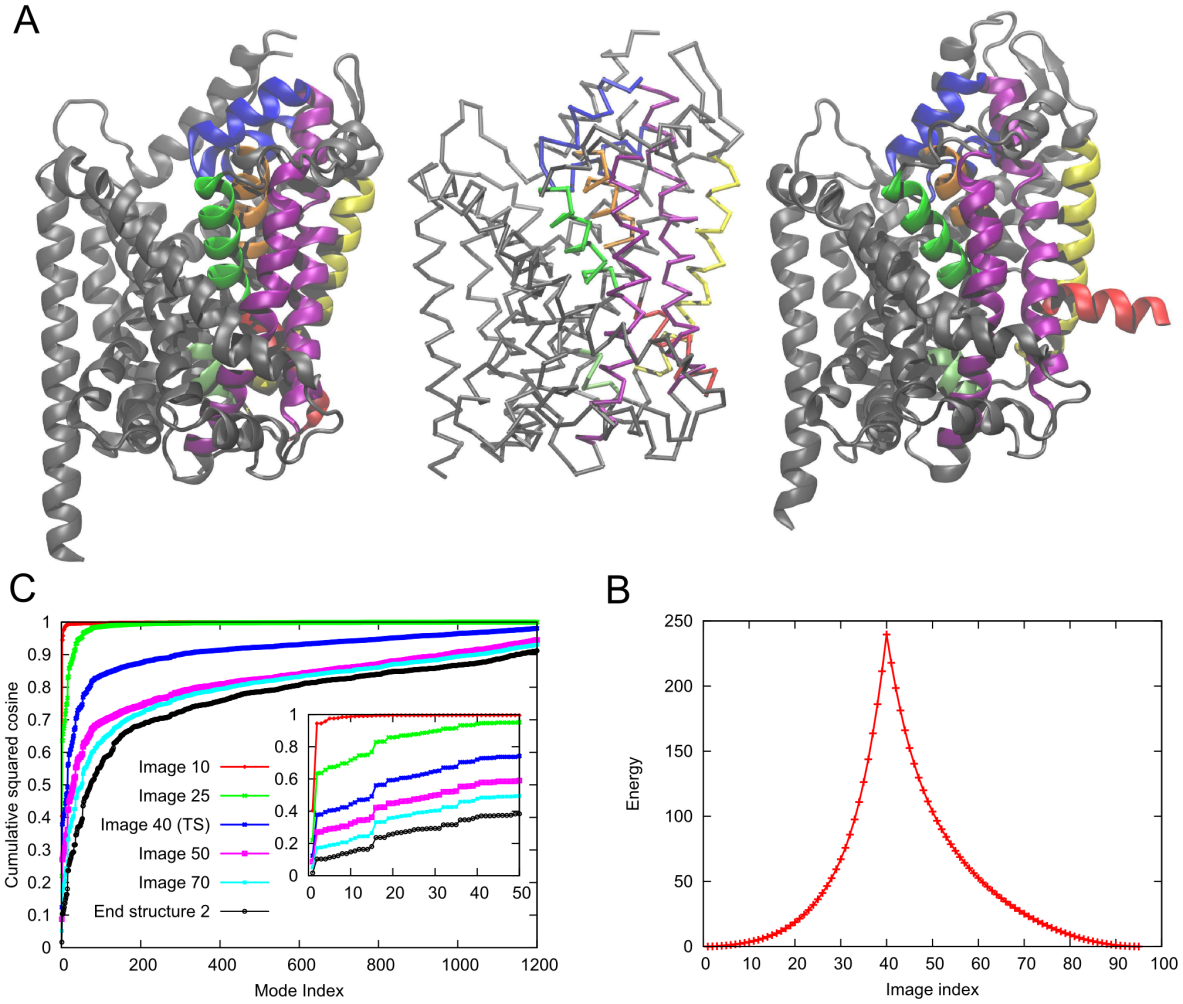
Conformational transition between the outward-facing occluded/closed (OFc) and the inward-facing open (IFo) states of leucine transporter, LeuT. **A.** Structures of the OFc state (*left*, PDB ID: 2A65) and the IFo state (*right*, PDB ID: 3TT3). The scaffold domain, which does not undergo significant conformational changes, is shown in *gray* and the rest of the protein is shown in *blue* (EL4), *red* (TM1a), *orange* (TM1b), *purple* (TM2 and TM7), *yellow* (TM5), *green* (TM6a) and *lime* (TM6b). The central structure is the 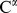 trace of the transition state produced by the *ANMPathway* method. **B.** The energy of the system along the transition. Total number of images in the pathway is 95, RMSD between two consecutive images is ∼0.05 Å. The transition state corresponds to image 40. **C.** Cumulative squared cosines between the structural change to reach a few selected conformers/images along the transition pathway and the ANM modes accessible to the starting (outward-facing occluded) structure.

Neurotransmitter sodium symporters (NSS) are integral membrane proteins responsible for secondary transport of glycine, *γ*-amino butyric acid and biogenic amines across the plasma membrane. LeuT is a bacterial orthologue of eukaryotic NSS. The protein consists of twelve transmembrane (TM) helices (TM1-12), extracellular (EC) (EL2, EL3, EL4a, El4b) and intracellular (IC) loops (IL1, IL5) and two *β*-sheets. The crystal structures of LeuT in the outward-facing (OF) occluded (PDB ID: 2A65 [73]) and inward-facing (IF) open (PDB ID: 3TT3 [74]) states have been resolved (left and right structures of Fig. (3A), see figure caption for color codes).

We note that several residues were not resolved in these structures. We have built the ANMs based on the residues commonly resolved in the two structures and used the scaffold region (TM3, TM4, TM8 and TM9) [74] for structural alignment of all conformers along the pathway.

The results are presented in Fig. (3B–C) for the transition from the OF occluded to IF open state. The pathway is composed of 95 images with an RMSD of 0.05 Å between two consecutive images and the transition state is located at image 40 (closer to the OF occluded state). In order to analyze the transition we have looked at the rearrangements of EL4a, EL4b, TM1a, TM1b, TM2, TM5, TM6a, TM6b and TM7 which play important roles in substrate-binding and gating at the EC and IC regions [74]. The motions are much more subtle compared to AK. At the initial stage there seems to be an almost rigid-body rotation in the aforementioned domains (movies S2 and S3 in SM). Then a downward movement of EL4a and EL4b closes the opening at the EC side. Subsequently, concerted motions of TM5 and TM1a occur. At the later phase of the transition, the main event is the movement of TM1a, which, along with other domains, creates an opening into the IC region. Barring the initial stage, various motions involve intra-domain movements and do not follow a strict rigid-body character. TM2 and TM7 move together for the entire duration of the conformational transition.

The complexity of the motions and lack of rigid-body character are reflected on the normal mode projections of various conformers along the pathway (Fig. (3C)). At the initial stage when the motions are rigid-body like, a few normal modes are sufficient to describe the structural change. But as the transition progresses many more normal modes are needed to represent the displacement from the reference (OF occluded) state. For the later stage of the transition and for the end-state almost 500 modes out of possible ∼1550 modes are required to attain a cumulative correlation of 0.8. This is in sharp contrast to the transition in AK where far fewer percentages of modes were sufficient in describing the structural changes.

**Figure 4:**
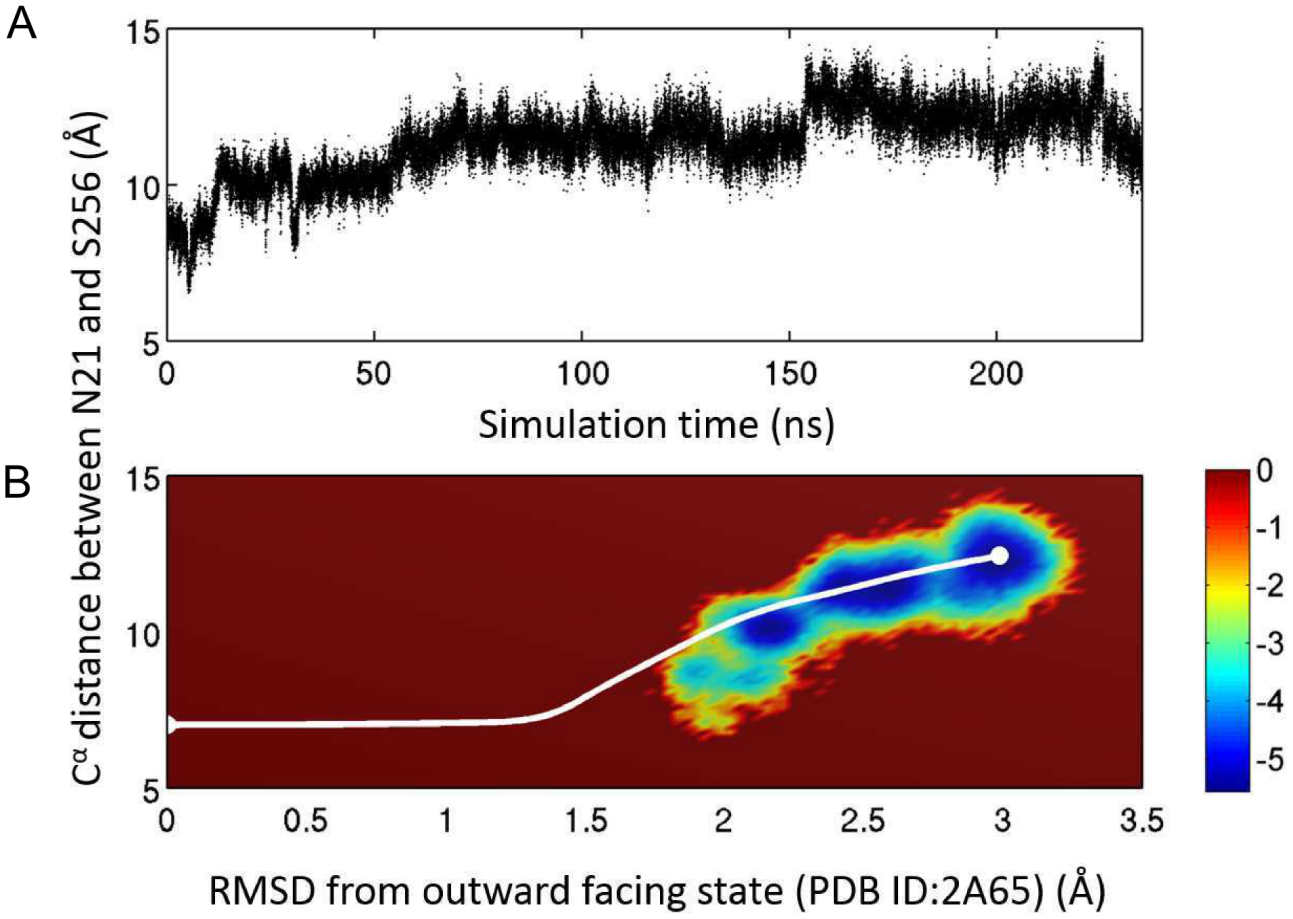
Comparison with all-atom simulation results. **A.** Time trace of the distance between 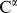 atoms of residues 21 and 256 from a 235 ns long conventional MD simulation of the fully solvated system. The simulation was started from a structure obtained from a targeted MD simulation originated from the OFc state. The system undergoes a spontaneous transition to the IFo state. **B.** Comparison of the *ANMPathway* method and all-atom MD in the space of two order parameters. The all-atom MD results are shown as a pseudo free energy landscape 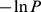, where *P* is the 2D distribution. The color-scale goes from *blue* (low energy) to *red* (high energy). The pathway predicted by *ANMPathway* (*white* line) goes mostly through the low energy regions of the free energy landscape.

To gain more insights and validate the analysis, we compared the pathway from *ANMPathway* to a 235 ns long all-atom MD trajectory using as initial structure an intermediate close to the OF occluded state [75]. The details of the simulation protocols are described in the text S1 of the Supplemental Material (SM). The time evolution of the structure during this transition was probed by monitoring a relevant order parameter, namely the 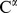 distance between the binding-site residues N21 and S256 shown in Fig. (4A). Notably, a spontaneous transition to IF open state was observed in this conventional (unbiased) full atomistic MD simulation. The MD trajectory thus provides an important data-set for benchmarking the *ANMPathway* method. Fig. (4B) compares the projection from the *ANMPathway* (white curve) and the MD trajectory on the space of two order parameters, the N21-S256 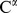 distance and the RMSD from the OF occluded state. The MD data are represented as a crude free energy calculated by taking the logarithm of the two-dimensional histogram of the above mentioned two order parameters shown in Fig. (4B). The pathway predicted by *ANMPathway* is constructed on a smooth potential energy function and has no thermal fluctuations. As such, it is representative of an average pathway of the real system and it should go through the low energy regions of the free energy landscape obtained from the MD simulation of LeuT embedded in fully solvated membrane lipids at finite temperature. This is exactly the behavior we observe in Fig. (4B) for the most parts where all-atom MD data are available. This agreement is satisfactory, given the minimal computational cost required by *ANMPathway* compared to that (several orders of magnitude larger) required for the full scale all-atom MD simulation.

**Figure 5:**
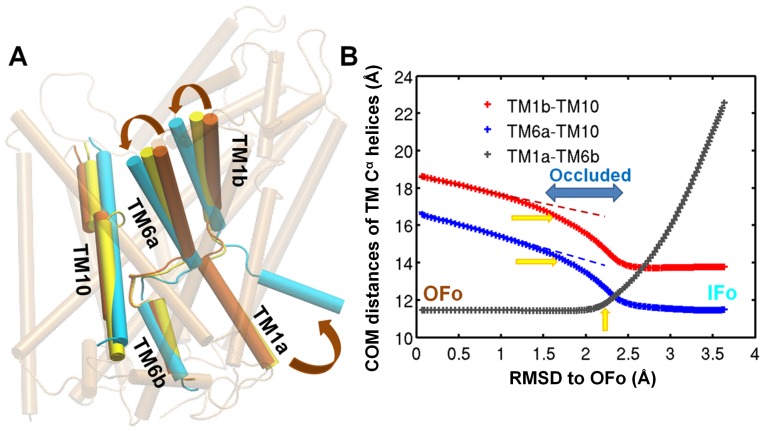
Occurrence of outward-facing occluded/closed (OFc) state along the computed transition pathway between outward-facing open (OFo) and inward-facing open (IFo) states of LeuT. **A.** Relative orientations of TM1, TM6 and TM10 of LeuT in the OFo (*orange*), OFc (*yellow*) and IFo (*cyan*) crystal structures. The OFo crystal structure is shown in transparent cartoon. Inward tilting of the TM1b and TM6a segments contributes to the closure of the extracellular vestibule, indicating the decreased distances of TM1b-TM10 and TM6a-TM10. Outward tilting of TM1a dominates the opening of the intracellular vestibule, resulting in the increased distance between TM1a andTM6b. **B.** Variation of the center of mass (COM) distances of TM1a-TM10 (*red*+), TM6b-TM10 (*blue*+), and TM1a-TM6b (*gray*+) as the LeuT undergoes transition from the OFo to IFo states. For COM distance calculations, TM1a (R11 to A22), TM1b (L25 to A35), TM6a (G242 to L255), TM6b (F259 to Y268), and TM10 (K398 to V412) 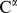 atoms are taken from *ANMPathway* calculations of LeuT from IFo to OFo. *Yellow arrows* point to the values found in the OFc crystal structure. Clearly, occluded intermediates are identified by *ANMPathway* calculations (highlighted by the horizontal *blue arrow*).

There is another crystal structure of LeuT which models the outward-facing open state (PDB ID: 3TT1 [74]). The sequence of functional states in the reaction cycle is: OF open (PDB ID: 3TT1)
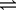
OF occluded (PDB ID: 2A65)
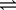
IF open (PDB ID: 3TT3). There are important differences in the helical orientations of several TM helices between the OF open and OF occluded structures (Fig. (5A)) even though their overall architectures are quite similar. It is natural to ask whether the *ANMPathway* could predict the existence of OF closed state along a transition pathway calculated between OF open and IF open states. We indeed found a conformer which is very close to the OF closed state (RMSD from 2A65: ∼1.0 Å) along the pathway between the crystal structures of OF open and IF open states. The detection of the occluded intermediate is studied by monitoring order parameters that describe the instantaneous conformations of the TM helices responsible for gating and binding of ions. The order parameters are the center of mass (COM) distances between the pairs of helices TM1a-TM10, TM6b-TM10 and TM1a-TM6b. The results are shown in Fig. (5B). In all three distance profiles the intermediate is detected as indicated by the yellow arrows. Given the simplicity of the potential energy function, it is quite remarkable that the method is capable of detecting a functionally relevant and experimentally observed intermediate state. This fact highlights the usefulness of the method as well as significance of global modes of motion in facilitating the conformational transition of transporters.

### Glutamate Transporter (Glt_Ph_)

**Figure 6:**
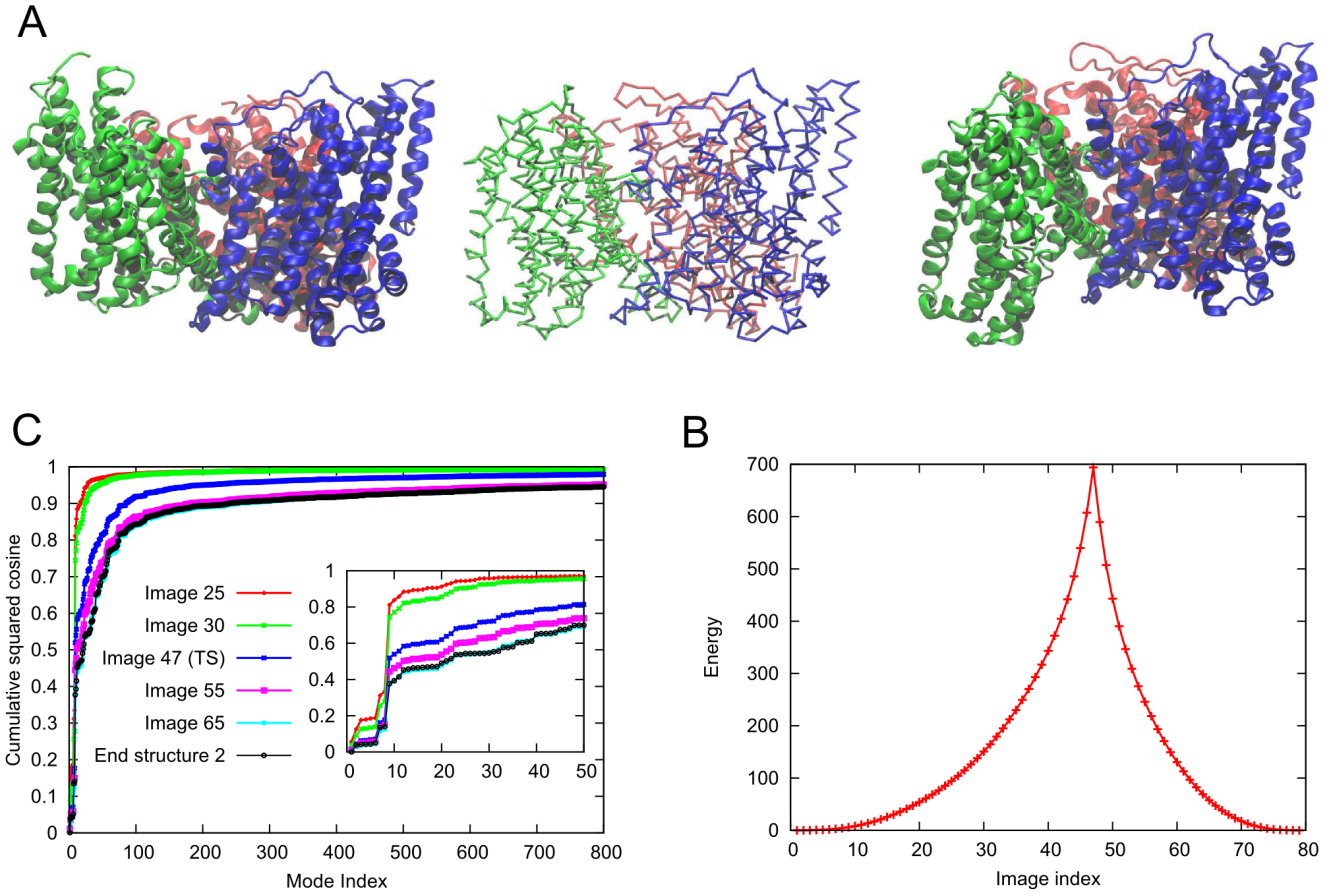
Conformational transition between states with two protomers facing inward and all protomers facing inward of the glutamate transporter, Glt_Ph_. **A.** *Left*: Structure of the intermediate state (iOF) state where two protomers are in IF and the third (*green*) protomer is in the OF conformations (PDB ID: 3V8G); *right*: IF state where all protomers are in IF conformations (PDB ID: 3KBC). The central structure is the 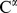 trace of the transition state produced by *ANMPathway*. **B.** The energy of the system along the transition. Total number of images in the pathway is 79, RMSD between two consecutive images is ∼0.1 Å. The transition state corresponds to image 47. **C.** Cumulative squared cosines between the structural change to reach a few selected conformers/images along the transition pathway and the ANM modes accessible to the starting (iOF) structure.

Excitatory amino acid transporters (EAATS) constitute a class of integral membrane proteins that are responsible for secondary active transport of amino acids like glutamate and aspartate across the plasma membrane. The aspartate transporter 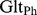, an archaeal orthologue of eukaryotic EAAT, is broadly used as a structural prototype, being functionally resolved in multiple states. The protein is a homotrimer. Each protomer consists of eight TM helices (TM1-8) and two helix-turn-helix motifs (HP1 and HP2) at the substrate-binding core [76]. According to the alternating access mechanism that enables the transport of substrate, the trimer alternates between OF and IF states and *vice versa*, via structural changes in all three protomers, to expose the substrate-binding site to the EC and IC regions, respectively. Crystal structures of 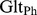 in OF (PDB ID: 1XFH [76]), IF (PDB ID: 3KBC [77]) states, as well a mixed intermediate state (iOF) with two protomers in IF conformation and one in an intermediate between OF and IF conformations (PDB ID: 3V8G [78]) have been determined (Fig. (6A)). The iOF state has been suggested to be relevant to uncoupled anion permeation during the transport process [78]. This asymmetric structure closely approximates the intermediate predicted earlier by a combined experimental and computational study [79].

We examined the transition between the iOF and IF states, using *ANMPathway*. The crystal structures reveal that protomers can be divided into two domains: a trimerization domain (TM1, TM2, TM4 and TM5) that closely maintains its internal conformation during the transition, and a transport domain (TM3, TM6, HP1, TM7, HP2 and TM8) that practically undergoes a downward rigid-body movement (perpendicular to the membrane) relative to the trimerization domain. Our pathway is made of 79 images with an RMSD of 0.1 Å between two consecutive images and the transition state is located at image 47 (Fig. (6B)). In accord with experimental data, the trimerization domain does not exhibit significant change in its internal structure during the entire transition while the transport domain of the reconfiguring protomer (colored green in Fig. (6A)) first moves like a rigid-body and then undergoes intra-domain rearrangements. The short segment of (the broken helix) TM8 moves first, then the lower longer segment and at the end the entire helix moves. Similarly, the upward part of TM7 moves initially followed by the rest of the helix. The movements of HP1 and HP2 are particularly important since they are involved in substrate gating [77]. Along the inward transition of the protomer, HP2 moves first followed by HP1 toward the end, consistent with the higher (initial) mobility of HP2 observed in all-atom MD simulations [80], [81]. See the movies S4 and S5 in SM. The long flexible loop in the extracellular part also undergoes significant movements during the transition. A simpler view of these local rearrangements can be obtained if one constructs two blocks as suggested by Reyes *et al.* [77] and predicted by ANM analysis to constitute two distinctive substructures subject to anticorrelated motions [82]. Block 1 is composed of HP1 and the lower part of TM7; and block 2 consists of HP2 and the upper part of TM8 up to the point where the helix is broken. Block 2 moves first followed by block 1 (movie S6 in SM).

Fig. (6C) displays the number of normal modes needed for describing the transition to several images/conformers starting from the iOF state. At the initial stage when motions are more rigid-body like, very few modes are sufficient to describe the structural changes. However, similar to LeuT, this number increases as more localized events that do not conform to *en bloc* movements of low frequency modes become important. These involve flexible regions within the transport domain. However, the total number of modes for a reasonable description of the overall structural change remains significantly smaller than those available (e.g. <100 modes accomplish a cumulative squared cosine of 0.8 with the reference state), supporting the utility of low frequency modes for efficiently mapping the transition pathway.

**Figure 7:**
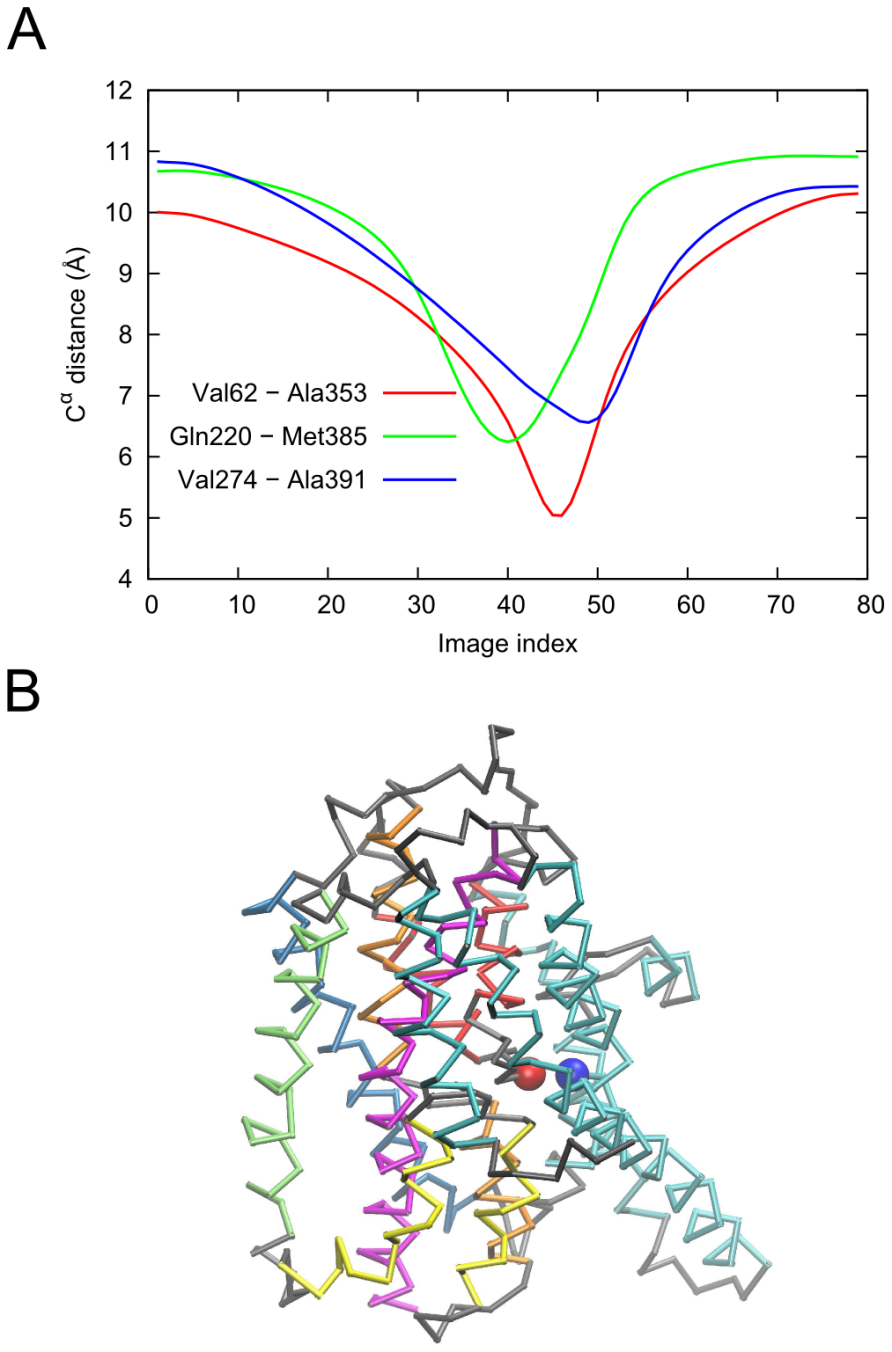
Occurrence of transient non-native contacts along the conformational transition of Glt_Ph_ between iOF and IF states. **A.** Distances of three contact-forming pairs plotted against the index of the conformers along the pathway. All contacts are formed between residues belonging to the same protomer. Two residues are said to form a non-native contact if they are more than 10 Å apart in the reference states but somewhere along the transition, the pairwise distance between them becomes less than 7 Å. **B.** Conformer at the point of closest contact between Val62 and Ala353 (lowest point of the *red* curve in **A**, corresponding to the image 46). The contact forming residues, Val62 and Ala353 are shown as *blue* and *red* spheres respectively. The color code is: trimerization domain, *cyan*; TM3, *deep sky blue*; TM6, *lime*; HP1, *yellow*; TM7, *orange*; HP2, *orange*; TM8, *magenta* and the rest, *gray*.

The complex structural changes result in the formation of eight non-native contacts along the transition. The formation of non-native contact is defined as two residues that are more than 10 Å apart in the end-states but come closer by less than 7 Å during the transition. The non-native contact forming pairs are Val58-Ala358, Val62-Ala353, Leu152-Leu347, Gln220-Met385, Val274-Ala391, Thr275-Gly357, Gly280-Pro356 and Val355-Ile361. All the pairs belong to the protomer that is undergoing the transition to the IF conformation (chain C, colored green in Fig. (6A)). It is worth noting that none of these pairs corresponds to the cysteine cross-link present in the crystal structure of the IF state [77]. Fig. (7) shows the distance profiles for three of these pairs along the transition. Most of these contacts involve pairs where one residue is in the trimerization domain and the other in the transport domain (movies S7 to S9 in the SM). These observations provide a route to test the predictions of the *ANMPathway* method against experiments.

**Figure 8:**
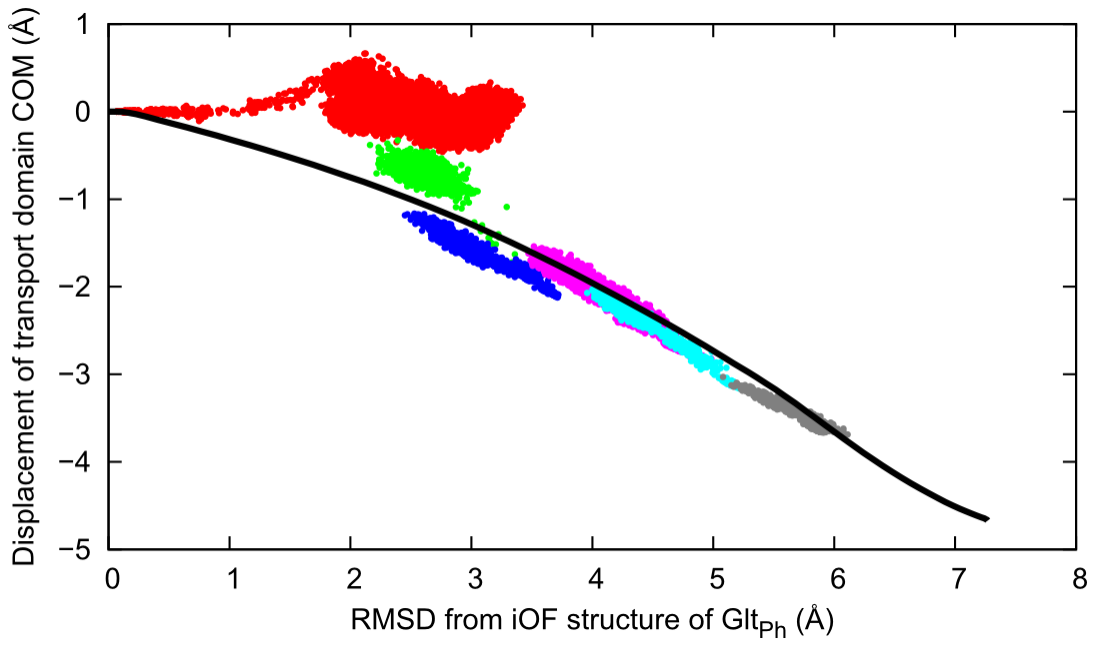
Comparison of *ANMPathway* with all-atom simulation results for the Glt_Ph_ transition. The *black* line is the projection of the pathway on a space spanned by two order parameters, RMSD from the endpoint (iOF state) and the *z* component of the displacement of the center of mass (COM) of the transport domain (based on 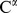 atoms) with respect to the initial crystal structure. Negative values along the ordinate point to the IC region. The dots of different colors are the projection of conformers sampled in unbiased all-atom MD simulation runs initiated from various points along a TMD path between the two end-states. For the middle section the data clouds from the unbiased MD simulations cluster around the pathway predicted by *ANMPathway*.

We have compared the *ANMPathway* results with the data collected from all-atom simulations. It is very challenging to simulate a spontaneous transition by straightforward conventional MD simulations. In order to perform a qualitative comparison, we have adopted the following protocol. First, a targeted MD (TMD) pathway is generated between the end-states (iOF and IF) with targeting forces acting on the backbone atoms only (see SM text S1 for details). Then, we launched a series of conventional MD runs from various intermediates visited during the TMD. These runs might be expected to follow the local free energy gradient and proceed along the result from *ANMPathway* provided that the latter offers a reasonable approximation to the actual transition pathway. In order to understand the transition in terms of a simple order parameters we have used, as order parameter, the *z* component of the distance vector between the COM of the 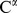 atoms of the transport domain of a conformer and that of the crystal structure of the iOF state. Fig. (8) shows the projection of the predicted pathway and snapshots from unbiased MD runs initiated from various intermediate structures (shown in different colors) on the space spanned by the above-mentioned order parameter and by the RMSD from the end structure (iOF state). Except for one of the trajectories (shown in red points, near the starting point), the MD runs yielded snapshots in accord with the transition pathway predicted by *ANMPathway*. The iOF state of 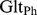 represents an intermediate between the OF and IF states, it is conceivable that the MD runs starting from this intermediate (shown in red) tend to go back to the more stable OF state, instead of drifting toward the IF state. This is primarily due to the proximity of the initial structure to the deep free energy basin of the more stable (OF) end-state. Overall, these data validate the ability of *ANMPathway* to provide a meaningful description of the structural changes involved in the global transition of 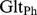 protomers as they reconfigure from OF to IF states.

### Sarcoplasmic Reticulum Ca^2+^-ATPase (SERCA)

**Figure 9:**
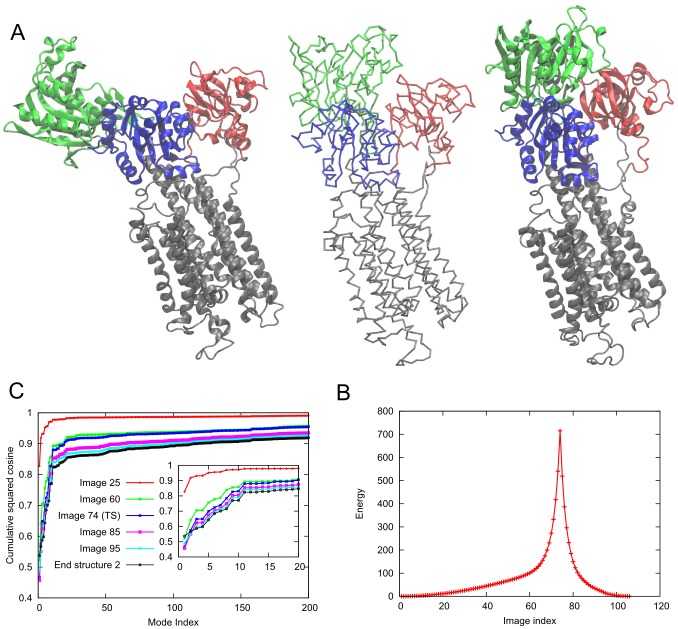
Conformational transition between E1.2CA^2+^ and E1.ATP states of the Sarcoplasmic Reticulum Ca^2+^-ATPase (SERCA). **A.** Structures of the 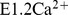 (*left*, PDB ID: 1SU4) and the E1.ATP (∼E1P) (*right*, PDB ID: 1T5S) states. The P, A and N domains are shown in *blue*, *red* and *green* respectively. Rest of the protein including the transmembrane domain is shown in *gray*. The central structure is the 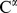 trace of the transition state produced by *ANMPathway*. **B.** The energy of the system along the transition. Total number of images in the pathway is 106, RMSD between two consecutive images is ∼0.2 Å. The transition state corresponds to image 74. **C.** Cumulative squared cosines between the structural change to reach a few selected conformers/images along the transition pathway and the ANM modes accessible to the starting (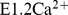 state) structure.

Calcium transporting pump of sarco/endoplasmic reticulum (SERCA) is an integral membrane protein that pumps 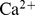 ions from calcium-poor cytoplasm of the muscle cell to the calcium-rich lumen of the sarcoplasmic reticulum at the expense of ATP hydrolysis. This process gets rid of the excess 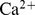 ions in the cytoplasm caused by their release from the lumen during muscle contraction and reverts the muscle to relaxed state. The protein is composed of a single polypeptide chain of 994 amino acids that form three cytoplasmic domains (nucleotide-binding domain N, phosphorylation domain P, actuator domain A) and ten TM helices (M1–10) (Fig. (9A)). Extensive structural studies have revealed atomic models of various functionally relevant states in the pumping cycle [83]. We have used the *ANMPathway* method to explore the transition between the 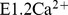 (PDB ID: 1SU4 [84]) and E1.ATP (PDB ID: 1T5S [85]) states. In the 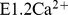 state the calcium ions can dissociate from the transmembrane binding sites but, in the E1P state (i.e. phosphorylated 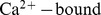 state) they are occluded and can not go back to the cytoplasmic side. The architecture of the E1.ATP state is almost identical to that of E1P or the transition state analog E1∼P.ADP state (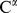 RMSD<0.5 Å). Therefore, at the level of a 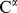 CG model, a transition pathway between the 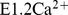 and E1.ATP states can provide important insights into the large scale motions responsible for occlusion of 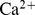 ions in the transmembrane binding sites.

The pathway consists of 106 images/conformers with an RMSD of 0.2 Å between two consecutive images and the transition state is located at image 74 which is closer to the E1.ATP state (Fig. (9B)). Careful examination of the pathway obtained by *ANMPathway* reveals that at the initial stage only the N domain moves while the rest of the protein remains fixed. This is reflected in the initial slow rise in the energy as shown in Fig. (9B). As the N domain comes closer to the P and A domains, first the P domain undergoes intra-domain changes and then the A domain rotates. This rotation causes upward motions of M1 and M2 and in this process M1 helix adopts a bent conformation. The energy rises quickly as the cytoplasmic domains close and local structural rearrangements take place. The bending of the M1 helix is responsible for shielding the TM calcium-binding sites from the cytoplasmic side and helps to form the occluded state [83], [86] (movie S10 in SM). These observations can be regarded as a crude qualitative picture of the conformational couplings among various parts of SERCA that give rise to the occluded state. The initial motion of the N domain which costs little energy suggests that the protein can exist in alternative conformations where the N domain may be much closer to other cytoplasmic domains compared to the 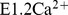 crystal structure. This observation is validated by other simulation and experimental studies [87], [88]. Fig. (9C) shows that only a handful of modes are sufficient for describing the transition. This is due to the initial rigid-body motion of the N domain, in good agreement with the slow modes predicted by the ANM based on the 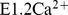 structure. The entire pathway can be projected onto ∼50 modes to take into account more than 80% of the displacement. The normal mode picture is therefore very useful for exploring the transition of this system.

**Figure 10:**
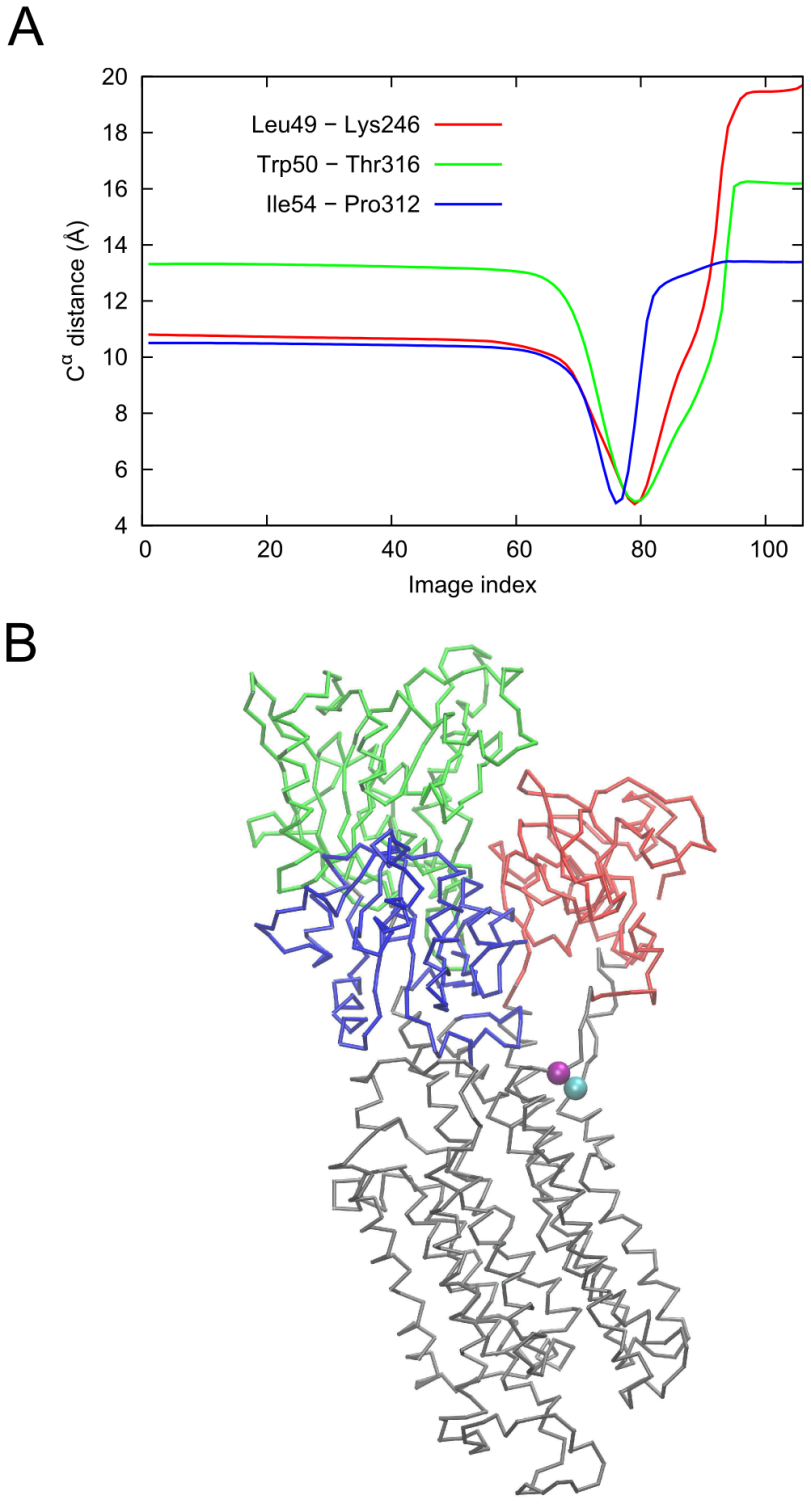
Occurrence of transient non-native contacts along the conformational transition of SERCA. **A.** Distances between contact-forming residue pairs plotted against the index of the conformers along the pathway. Two residues are said to form non-native contact if they are more than 10 Å apart in the reference states but somewhere along the transition, the pairwise distance between them becomes less than 5 Å. **B.** Intermediate conformer at the point of closest contact between Leu49 (cyan sphere) and Lys246 (*purple* sphere) (minimum of the red curve in **A**, corresponding to the image index 79). The color code is same as in Fig. (9).

The distance profiles of contact-forming residues along the transition are shown in Fig. (10). The formation of non-native contact is defined as two residues that are more than 10 Å apart in the reference states but come close by less 5 Å during the transition. All contact-forming pairs involve one residue in the M1 helix. The other residue is either on the M4 helix or on the cytoplasmic loop region (see movies S11 to S13 in the SM). These contacts form as the result of bending of the M1 helix which is thought to be responsible for the formation of the occluded state. Therefore the non-native contacts can be probed experimentally to validate the predictions of method as well as to establish structural changes that have functional consequence.

## Discussion

We presented a new computational method, *ANMPathway*, for constructing the most probable (lowest energy) transition pathway between two stable endpoints of a conformational transition. Conceptually, *ANMPathway* represents a direct application of the string method to a two-state CG system approximated by ANM energy surfaces. For this reason, the method is simple and efficient; it can produce a completely optimized pathway for a 1000 residue protein in about one hour on a single CPU of a standard desktop computer. We have implemented the method as an open access web server with user-friendly features at http://anmpathway.lcrc.anl.gov/anmpathway.cgi. Although there exist other servers for exploring the transition paths between pairs of known endpoints [53], [55], [61], [89]–[91], *ANMPathway* is perhaps one of the simplest approaches that efficiently provides a unique solution for the most probable pathway, with a minimal number of parameters and no biased simulations using the ANMs for the two endpoints. The resulting pathway can be interpreted as the minimum energy pathway on the two-state elastic network surface with a cusp hypersurface. The presence of cusp hypersurface does not seem to influence the qualitative nature of the pathway as evident from the comparison of our results on adenylate kinase with those from other methods. Franklin *et al.* [61] in their MinActionPath method effectively used a similar two-state potential with cusp hypersurface and their results for the AK system are in good agreement with ours. We note that the presence of cusp hypersurface will have noticeable effects on the quantitative details of the pathway, especially near the transition state region. The energetic cost of breaking non-bonded contacts increases as one moves away from the native state which is reflected in the rapid change in energy near the transition state. A smooth surface, like the one adopted in other similar studies [52], [55], [89], will have a better representation of the underlying physics compared to our present two-state potential with a cusp hypersurface. This is the sacrifice we have to make in order to exploit the simplicity and algorithmic efficiency imparted by the two-state potential. However, the influence of this drawback on the pathways produced by *ANMPathway* was verified to be minimal and localized in the transition state region. This is clearly demonstrated by the refinement of the pathways by systematic smoothing of the two-state potential as described below. The method of Zheng *et al.* [55] had been implemented as a web server called AD-ENM which allowed us to directly compare our results with that of a similar method based on a more realistic energy surface. The pathways produced by the AD-ENM server had different numbers of images than the corresponding pathways generated by *ANMPathway*. For the purpose of comparison, we projected the pathways using the same sets of order parameters already used to analyze *ANMPathway* results in the previous section. For the AK case, it seems that AD-ENM produced a qualitatively different pathway than *ANMPathway* (SM Fig. S1). The AD-ENM pathway is closer to the scenario (iii) in Fig. (2), whereas *ANMPathway* result resembles scenario (i) in the same figure. Both methods produced qualitatively similar pathways for LeuT and 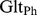 as illustrated by the projections presented in SM Figs. S2 and S3 respectively. The AD-ENM server makes small modifications to the end-states which can explain the discrepancy found near one of the endpoints in SM Fig. S2. The *ANMPathway* method is also closely related to the PNM method of Maragakis and Karplus [52]; our two-state potential is a limiting form of their smooth two-state ENM, when the mixing parameter goes to zero. We use a novel and efficient algorithm for searching for the minimum energy structure on the cusp hypersurface, which is equivalent to the saddle point search in the PNM method. If a sufficiently small value of mixing parameter is used, the PNM result will be very similar to ours and this is indeed observed for the AK system.

**Figure 11:**
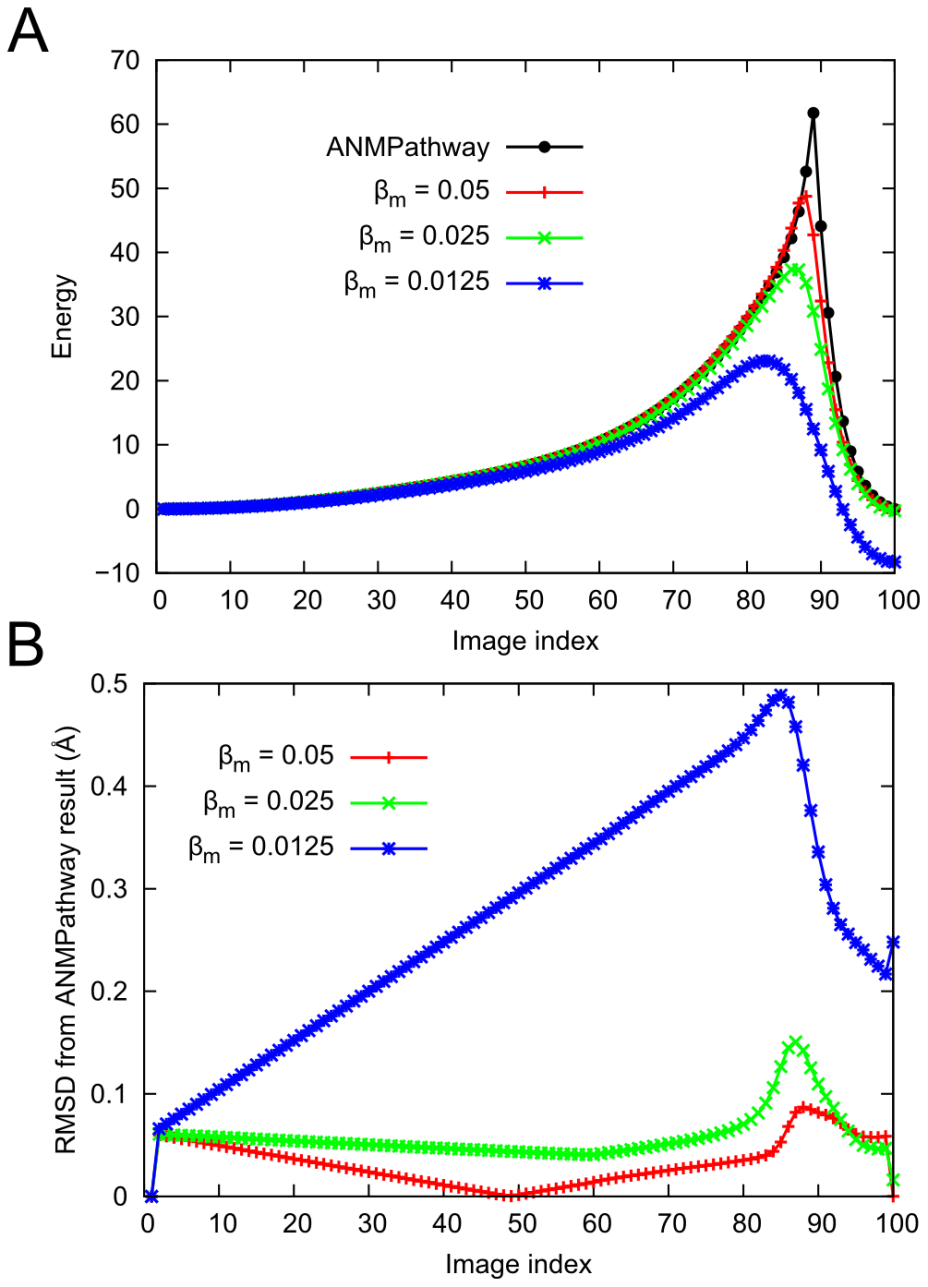
Effect of smoothing of the two-state potential on the adenylate kinase transition pathway. **A.** Energy of the system along the pathway for several pathways obtained by refining the original pathway by zero temperature string method calculation on the smoothed potential defined in Eq. (9). **B.** RMSD of the refined pathways from the pathway produced by *ANMPathway*.

The effect of the cusp hypersurface on the potential energy surface was further examined by refining the pathway on a smoothed surface based on the following potential function [54], [57],
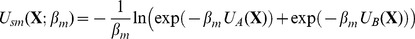
(9)where 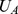 and 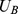 are ANM potentials defined in Eq. (1). The parameter 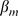 determines the amount of mixing and the height of the barrier. In the limit 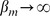, the potential defined above becomes the two-state potential with a cusp hypersurface defined previously in Eq. (4) i. e. 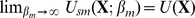. Therefore, if we refine the pathway after smoothing the potential using Eq. (9) with a high value of 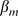, the resulting pathway should not be very different. We tested this hypothesis by performing zero temperature string method [92] calculation on the smoothed energy surface starting from the final path produced by the *ANMPathway* method. The results for AK are shown in Fig. (11). It is noteworthy that, for 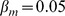, the RMSDs between corresponding images along the two pathways are extremely small (compared to the the resolution of the structures) and the position of the transition state is almost unchanged (the projections of the pathways on the space spanned by the two order parameters described in Fig. (2) are shown in the SM Fig. S4). As the value of 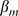 is decreased the RMSDs increase and the position of the transition state moves slowly to the right. These observations demonstrate that the algorithm used in *ANMPathway* has the ability to find a proper transition state on the two-state surface with the cusp hypersurface and also illustrate that presence of cusp hypersurface does not have a significant impact on the over all features of the two-state potential.

A desirable feature of any pathway method is the ability to detect functionally relevant intermediate structures. The two-state potential energy function used in the *ANMPathway* may be too simple for such purpose. If an intermediate has a topology which is very different from both the end-states then it is unlikely to be detected by this method. However, it is reasonable to expect that an intermediate that maintains the overall fold and much of the secondary structure shared by the two endpoints would show up along the computed transition pathway. This was verified in the transition pathway of LeuT from OF open to IF open state, via the intermediate OF occluded state (see Fig. (5)) consistent with experimental data. We note that Adelman *et al.* [93] also observed the passage over an occluded intermediate in their weighted-ensemble simulations of the transition of Mhp1 (another transporter that shares LeuT fold) between its OF and IF states. In their case, a significantly more detailed two-state G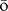 potential (with several parameters) is used for each endpoint, together with a computationally expensive Monte Carlo sampling algorithm (compared to *ANMPathway*). The present approach offers the multiple advantages of being simpler and more efficient, while detecting at the same level of resolution an experimentally validated intermediate.

The effective two-state CG potential used by *ANMPathway* is meant to approximate the true PMF of the system with respect to the 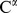 coordinates chosen as collective variables [14]. The basic assumption of such structure-based CG models is that the information about the native contact topology is sufficient for describing large scale transitions. Yet, the effective CG potential can produce pathways with various degrees of complexity depending on the system. For AK, the main structural difference between the end-states is rigid movements of large domains and the transition also involves largely rigid-body motions of various domains. But for 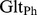, even though the inspection of end-states reveals a rigid-body movement of the transport domain with respect to the trimerization domain, the structural changes along the pathway are much more complex. This fact highlights that simple ANM-based surfaces are capable of capturing significant complexity of biomolecular conformational change. For systems involving large-scale rigid-body domain motions, a few ANM modes predicted for the initial structure enable displacements far away from the starting state toward the end-state, as observed in transitions in AK and SERCA. However, if the intermediate structures involve more subtle changes, including localized motions, many more modes may be necessary (e.g. the case of LeuT). Notably, ANM-based surfaces are capable of capturing the complexity of biomolecular conformational change by inclusion of a higher number of modes. These observations validate the conventional picture of correlation between initial direction of structural change and displacements along low frequency normal modes [42]–[44] and will have important ramifications for pathway methods based on subsets of normal modes.

From a computational point of view, *ANMPathway* can be used as a first approximation to simulate the real transition in all-atom models. Our simple CG model does not have any residue-specific information or side chains and for membrane protein systems we introduce a further simplification by excluding the membrane. Therefore, results from *ANMPathway* should be interpreted accordingly and should only be used for a quick assessment of a likely path, or for studying relatively large-scale movements such as those of entire domains or subunits. The study demonstrates that *ANMPathway* can yield very good starting point for building all-atom pathways and refining them with more advanced methods.

Experimental validation of any conformational transition pathway produced via computations is obviously difficult. Owing to their stability, the end-states are often amenable to direct observation by scattering or spectroscopic methods. In contrast, the short-lived intermediate structures occurring transiently along the pathway are much more difficult to detect directly. The most one can hope for is that the predicted pathway could be validated indirectly. In this regard, a particularly interesting possibility is to engineer cross-links between pairs of residues that come in close contact somewhere along the pathway but are otherwise far away from one another in the two end-states. In practice, we were able to find such pairs of residues in 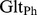 and in SERCA which are farther than 10 Å in both end-states, but transiently come within 5–6 Å from one another along the transition pathway. This is encouraging and perhaps experimentally probing pairs of residues satisfying this criterion could become a routine endeavor to validate and test computational pathways.

## Supporting Information

Figure S1 Comparison of AD-ENM [55] and *ANMPathway* paths of AK.
(PDF)

Figure S2 Comparison of AD-ENM [55] and *ANMPathway* paths of LeuT.
(PDF)

Figure S3Comparison of AD-ENM [55] and *ANMPathway* paths of 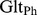.(PDF)

Figure S4 Projection of refined pathways on the smooth two-state potential defined in Eq. (9) on the space of order parameters used in Fig. (2).
(PDF)

Text S1Protocols for all-atom molecular dynamics simulations of LeuT and 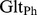.(PDF)

Video S1 Transition pathway between open and closed states of AK.
(MP4)

Video S2 Transition pathway between the outward-facing occluded state and inward-facing open state of LeuT.
(MP4)

Video S3 Structural changes in functionally important domains of LeuT. For clarity only the EL4a (*blue*), EL4b (*blue*), TM1a (*red*), TM1b (*orange*), TM2 (*purple*), TM5 (*yellow*), TM6a (*green*), TM6b (*lime*) and TM7 (*purple*) domains are shown.
(MP4)

Video S4Transition pathway between iOF and IF states of 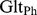. All three protomers are shown, the *green* protomer undergoes transition.(MP4)

Video S5Only the protomer undergoing transition in 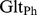 is shown with the following coloring scheme: trimerization domain, *cyan*; TM3, *deep sky blue*; TM6, *lime*; HP1, *yellow*; TM7, *orange*; HP2, *orange*; TM8, *magenta* and the rest, *gray*.(MP4)

Video S6A simpler view of the transition in 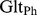 in terms of two blocks. The color code is: block 1, *blue*; block 2, *green*; rest of TM7, *orange*; rest of TM8, *magenta*.(MP4)

Video S7Contact formation between Val62 (*blue* sphere) and Ala353 (*red* sphere) of chain C during the transition between iOF and IF states of 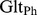.(MP4)

Video S8Contact formation between Gln220 (*blue* sphere) and Met385 (*red* sphere) of chain C during the transition between iOF and IF states of 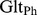.(MP4)

Video S9Contact formation between Val274 (*blue* sphere) and Ala391 (*red* sphere) of chain C during the transition between iOF and IF states of 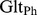.(MP4)

Video S10Transtion pathway between 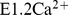 and E1.ATP states of SERCA.(MP4)

Video S11Contact formation between Leu49 (*cyan* sphere) and Lys246 (*purple* sphere) during the transition between 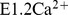 and E1.ATP states of SERCA.(MP4)

Video S12Contact formation between Trp50 (*cyan* sphere) and Thr316 (*purple* sphere) during the transition between 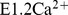 and E1.ATP states of SERCA.(MP4)

Video S13Contact formation between Ile54 (*cyan* sphere) and Pro312 (*purple* sphere) during the transition between 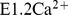 and E1.ATP states of SERCA.(MP4)
