# Correction: Exploring the Conformational Transitions of Biomolecular Systems Using a Simple Two-State Anisotropic Network Model

**DOI:** 10.1371/journal.pcbi.1003868

**Published:** 2014-08-29

**Authors:** The PLOS Computational Biology Staff

In the Author Contributions section, Avisek Das, Mert Gur and Mary Hongying Cheng are listed as having contributed equally to this work. Only Mert Gur and Mary Hongying Cheng contributed equally.
