# Supplementary material for: Exploring the Conformational Transitions of Biomolecular Systems Using a Simple Two-State Anisotropic Network Model
Source: PLoS Comput Biol. 2014 Apr 3;10(4):e1003521. doi: 10.1371/journal.pcbi.1003521 (PMC3974643; doi:10.1371/journal.pcbi.1003521)
Supplement: Figure S1 — Comparison of AD-ENM [55] and ANMPathway paths of AK. (PDF) [file pcbi.1003521.s001.pdf]

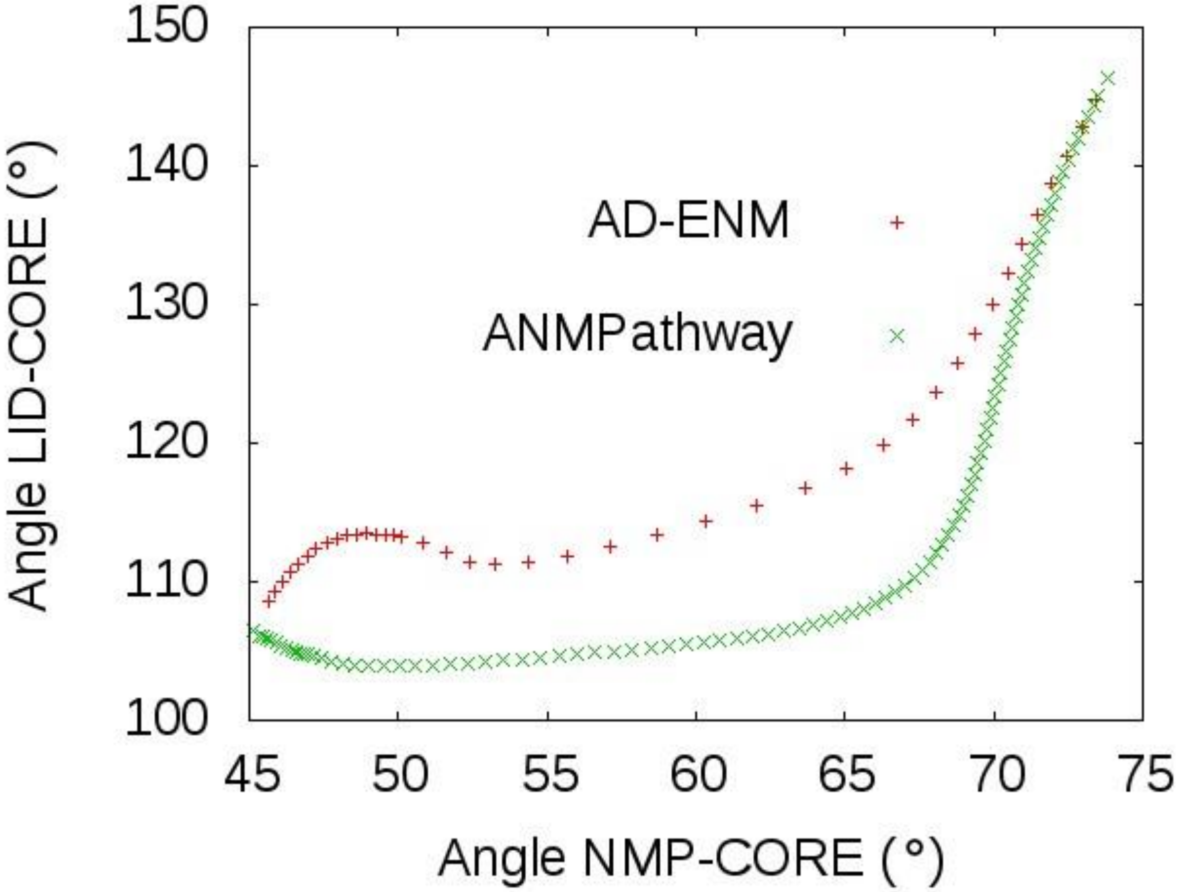

Figure S1. Comparison of *AD-ENM* and *ANMPathway* paths of AK. Projections of pathways on the space spanned by LID-CORE and NMP-CORE angles. See caption of Figure 2 in the main manuscript for angle definitions.
