# Supplementary material for: Exploring the Conformational Transitions of Biomolecular Systems Using a Simple Two-State Anisotropic Network Model
Source: PLoS Comput Biol. 2014 Apr 3;10(4):e1003521. doi: 10.1371/journal.pcbi.1003521 (PMC3974643; doi:10.1371/journal.pcbi.1003521)
Supplement: Figure S2 — Comparison of AD-ENM [55] and ANMPathway paths of LeuT. (PDF) [file pcbi.1003521.s002.pdf]

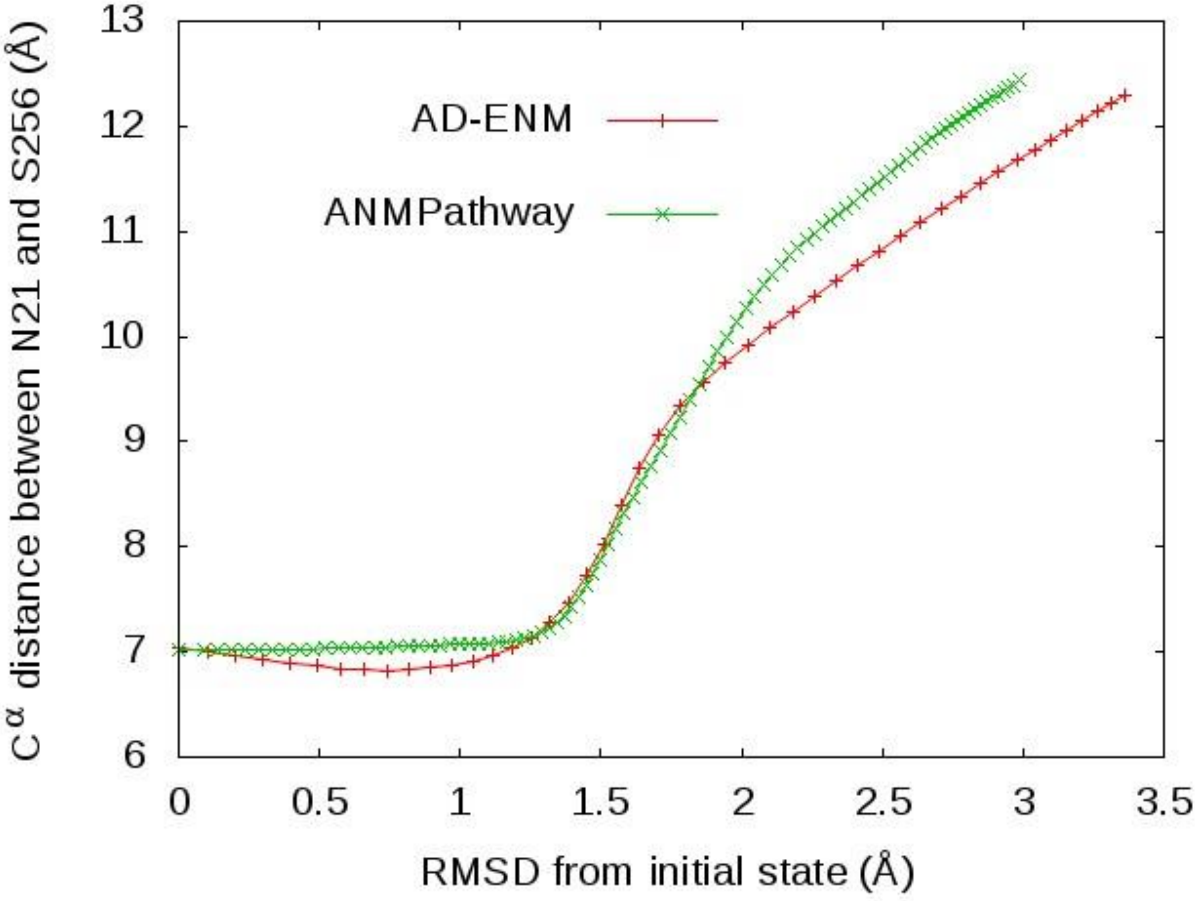

Figure S2. Comparison of *AD-ENM* and *ANMPathway* paths of LeuT. Projections of pathways on the space spanned by distance between C^{α} atoms of residues N21 and S256 and RMSD from the initial state.
