# Supplementary material for: Exploring the Conformational Transitions of Biomolecular Systems Using a Simple Two-State Anisotropic Network Model
Source: PLoS Comput Biol. 2014 Apr 3;10(4):e1003521. doi: 10.1371/journal.pcbi.1003521 (PMC3974643; doi:10.1371/journal.pcbi.1003521)
Supplement: Figure S3 — Comparison of AD-ENM [55] and ANMPathway paths of . (PDF) [file pcbi.1003521.s003.pdf]

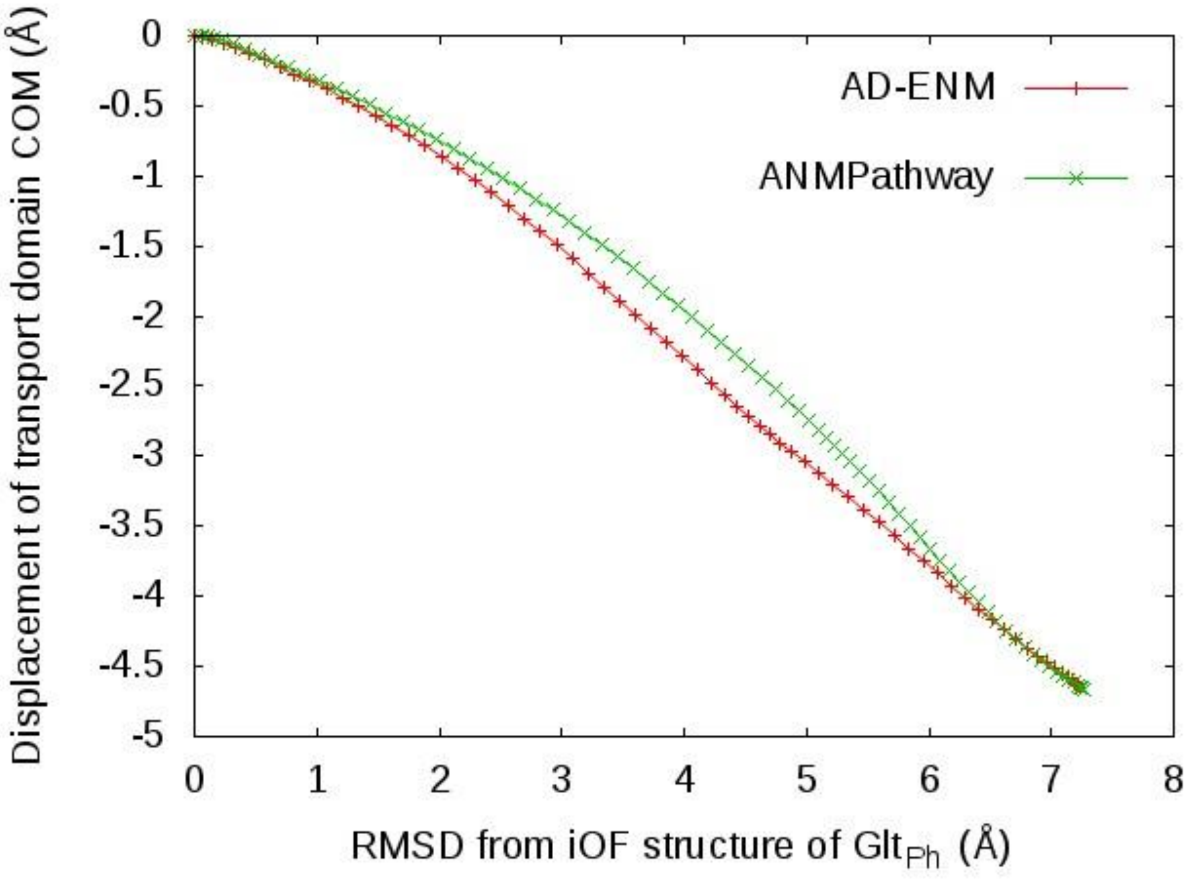

Figure S3. Comparison of *AD-ENM* and *ANMPathway* paths of Glt_{Ph}. Projections of pathways on a space spanned by two order parameters, RMSD from the end point (iOF state) and the *z* component of the displacement of the center of mass (COM) of the transport domain (based on C^{α} atoms) with respect to the initial crystal structure.
