# Supplementary material for: Exploring the Conformational Transitions of Biomolecular Systems Using a Simple Two-State Anisotropic Network Model
Source: PLoS Comput Biol. 2014 Apr 3;10(4):e1003521. doi: 10.1371/journal.pcbi.1003521 (PMC3974643; doi:10.1371/journal.pcbi.1003521)
Supplement: Figure S4 — Projection of refined pathways on the smooth two-state potential defined in Eq. (9) on the space of order parameters used in Fig. (2). (PDF) [file pcbi.1003521.s004.pdf]

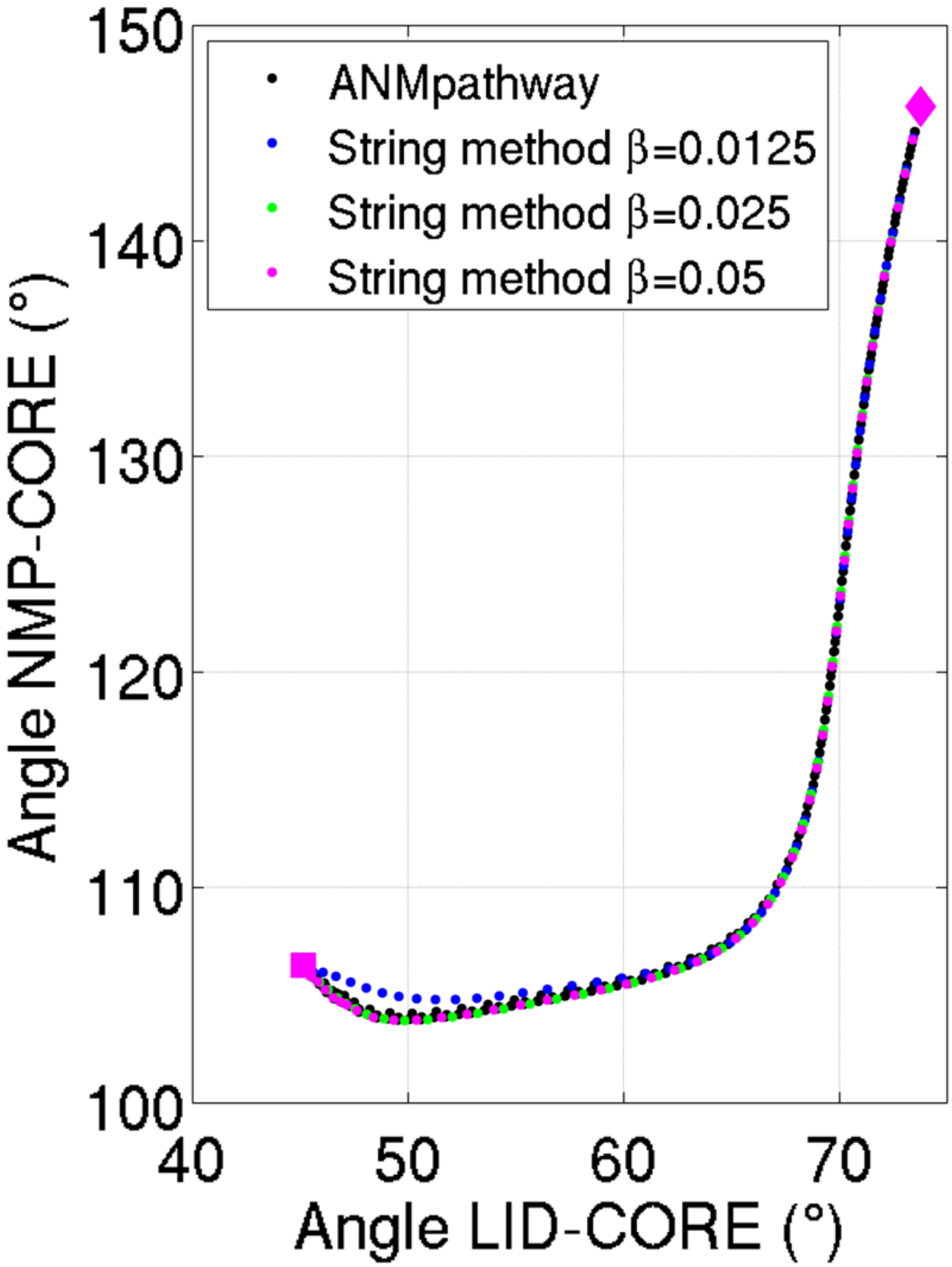

Figure S4. *ANMPathway* and string method paths of AK. Transition pathways displayed as a function of LID-CORE and NMP-CORE angles. Closed (bottom, square) and Open (top, diamond) substates of AK. String method paths are constructed by performing zero temperature string method calculation on the smoothed energy surface starting from the path produced by the ANMPathway method.
