# Supplementary material for: Exploring the Conformational Transitions of Biomolecular Systems Using a Simple Two-State Anisotropic Network Model
Source: PLoS Comput Biol. 2014 Apr 3;10(4):e1003521. doi: 10.1371/journal.pcbi.1003521 (PMC3974643; doi:10.1371/journal.pcbi.1003521)
Supplement: Text S1 — Protocols for all-atom molecular dynamics simulations of LeuT and . (PDF) [file pcbi.1003521.s005.pdf]

Molecular dynamics simulations. Conventional MD (cMD) simulations of intermediates deduced from targeted MD (tMD) simulations were carried out to investigate structural transitions from different states. In tMD simulation, a steering force of the form F_{tMD} = ½ (k/N) [(RMSD(t) – RMSD*(t)] was adopted, with the spring constant k = 200 kcal/(mol.Å^2); N is the number of targeted atoms, RMSD(t) is the instantaneous departure from the target crystal structure and RMSD*(t) is the target based on a linear decay from RMSD(0) to zero. For the cMD run, the system was first energy minimized for 20,000 steps, followed by an equilibration of 2 ns during which the backbone constraints (of 10 kcal/(mol.Å^2) on protein were gradually removed. Productive MD runs (unrestrained Nosé-Hoover (1, 2)) were performed at constant pressure (1 bar) and temperature (310 K). CHARMM36 force field with CMAP corrections was used for protein, water molecules, and lipids (3, 4).

Simulation protocols included periodic boundary conditions, water wrapping, hydrogen atoms constrained via SHAKE, and evaluation of long-range electrostatic forces via the Particle Mesh Ewald (PME) algorithm (5). The bonded and short-range non-bonded interactions were calculated at every time-step (2 fs), and electrostatic interactions were calculated every 4 fs. The cutoff distance for non-bonded interactions was 12 Å. A smoothing function was employed for the van der Waals (vdW) interactions at a distance of 10 Å. The non-bonded interactions list was updated every 20 time-steps for pairs within a distance cutoff of 13.5 Å.

System preparations. The missing loops in the crystal structures were re-constructed and refined using MODELLER 9.10 (6). All mutated residues in the crystal structures were mutated back to their wild-type amino acid residues. Protonation states of titratable residues were assigned based on pKa calculations using PROPKA (7). Then the transmembrane domain of the protein was inserted into the center of a pre-equilibrated POPC membrane. Fully equilibrated TIP3 waters and 0.1 M NaCl were added to neutralize the system.

Transition from OF to IF in LeuT. Crystal structures corresponding to the outward-facing closed (OF*c**) (PDB: 2A65) (8) and inward-facing open (IF*o*) (PDB: 3TT3) (9) state of LeuT were prepared using VMD (10), following our previous study (11). LeuT simulation systems contain the same materials: a LeuT monomer, two Ala substrates, 30 Na^+, 35 Cl^-, 212 POPC, and about 16,770 water molecules to add up to about 86,900 atoms, with a simulation box of 100×100×96 Å^3.

A 10-ns targeted MD (tMD) simulation (tMD_OF*c**→IF*o*) was performed to drive structural transitions of LeuT from the OF*c** (PDB: 2A65) to IF*o* (PDB: 3TT3) state (11). One cMD simulation using an intermediate (~7.4ns) deduced from the tMD simulation (tMD_OF*c**→IF*o*) was performed for 240 ns, during which both substrate and sodium ions were released.

Transition from iOF to IF in Glt_{ph}. Crystal structures of Glt_{ph} corresponding to an asymmetric intermediate (PDB: 3V8G) (12) and symmetric inward-facing conformation (PDB: 3KBC) (13) were used in the simulations. This asymmetric structure (12) has two protomers in an inward facing state (IFS) and the third (chain C) in an intermediate conformation between the outward- and inward-facing states (iOFS). Glt_{ph} simulation system contains one Glt_{ph} trimer, 295 POPC, and about 27,766 water molecules for a total of over 141,883 atoms in a simulation box of 135 Å×135 Å×95 Å. Fig. S1 illustrates MD setup of the initial Glt_{ph} MD system.

A 12-ns targeted MD (tMD) simulation (tMD_*i*OF→IF) was performed to drive structural transitions of Glt_{ph} from *i*OF state (12) (chain C of PDB 3V8G) to IF (chain C of PDB 3KBC) state (13) . Five cMD simulations using intermediates deduced at 0ns, 2ns, 4ns, 6ns, and 8ns of tMD simulation (tMD_*i*OF→IF) were performed for 150 ns, 50 ns, 50 ns, 50 ns, and 40 ns.

References:

1. Nosé, S. 1984. A Unified formulation of the constant-temperature molecular-dynamics methods. *J Chem Phys*. 81:511-519.
2. Hoover, W. 1985. Canonical dynamics: equilibrium phase-space distributions. *Phys. Rev. A.* 31:1695.
3. MacKerell, A.D., Bashford, D., Bellott, M., Dunbrack, R.L., Evanseck, J. D., Field, M. J., Fischer, S., Gao, J., Guo, H., Ha, S., Joseph-McCarthy, D., Kuchnir, L., Kuczera, K., Lau, F.T.K., Mattos, C., Michnick, S., Ngo, T., Nguyen, D. T., Prodhom, B., Reiher, W.E., Roux, B., Schlenkrich, M., Smith, J. C., Stote, R., Straub, J., Watanabe, M., Wiorkiewicz-Kuczera, J., Yin, D., Karplus, M. 1998. All-atom empirical potential for molecular modeling and dynamics studies of proteins. *J. Phys. Chem. B* 102:3586–3616.
4. Klauda, J.B., R.M. Venable, J.A. Freites, J.W. O'Connor, D.J. Tobias, C. Mondragon-Ramirez, I. Vorobyov, A.D. MacKerell, Jr., and R.W. Pastor. 2010. Update of the CHARMM All-Atom Additive Force Field for Lipids: Validation on Six Lipid Types. *J Phys Chem B.* 114 7830-7843.
5. Darden, T., D. York, and L. Pedersen. 1993. Particle Mesh Ewald - an N.Log(N) Method for Ewald Sums in Large Systems. *J Chem Phys*. 98:10089-10092.
6. Fiser, A. and A. Sali. 2003. ModLoop: automated modeling of loops in protein structures. *Bioinformatics*. 19:2500-1.
7. Li, H., A.D. Robertson, and J.H. Jensen. 2005. Very Fast Empirical Prediction and Interpretation of Protein pKa Values. *Proteins*. 61.
8. Yamashita, A., S.K. Singh, T. Kawate, Y. Jin, and E. Gouaux. 2005. Crystal structure of a bacterial homologue of Na^{+}/Cl^{-}-dependent neurotransmitter transporters. *Nature*. 437:215-23.
9. Krishnamurthy, H. and E. Gouaux. 2012. X-ray structures of LeuT in substrate-free outward-open and apo inward-open states. *Nature*. 481:469-74.
10. Humphrey, W., A. Dalke, and K. Schulten. 1996. VMD: visual molecular dynamics. *J. Mol. Graph.* 14:33-38.
11. Cheng, M.H. and I. Bahar. 2013. Coupled global and local changes direct substrate translocation by neurotransmitter-sodium symporter ortholog LeuT. *Biophys J*. 105:630-639.
12. Verdon, G. and O. Boudker. 2012. Crystal structure of an asymmetric trimer of a bacterial glutamate transporter homolog. *Nat Struct Mol Biol*. 19:355-7.
13. Reyes, N., C. Ginter, and O. Boudker. 2009. Transport mechanism of a bacterial homologue of glutamate transporters *Nature*. 462:880-885
